# Evaluation and implementation of an independent Kilovoltage X‐ray‐based imaging platform for carbon ion radiotherapy

**DOI:** 10.1002/acm2.70501

**Published:** 2026-02-09

**Authors:** Yixiao Guo, Zhiqiang Liu, Qingzhen Zhu, Ming Cai, Hongyi Cai, Ruifeng Liu, Qiuning Zhang, Zhiguo Xu

**Affiliations:** ^1^ Institute of Modern Physics Chinese Academy of Sciences Lanzhou Gansu China; ^2^ University of Chinese Academy of Sciences Beijing China; ^3^ Gansu Provincial Hospital Lanzhou Gansu China; ^4^ LANZHOU ION THERAPY CO., LTD Lanzhou Gansu China; ^5^ Shanghai Shinetone Medical Equipment Co., Ltd Shanghai China; ^6^ Heavy Ion Medicine Center Wuhan University & Peopleʼs Hospital of Wuhan Economic and Technological Development Zone Wuhan Hubei China

**Keywords:** carbon‐ion radiotherapy, cone‐beam computed tomography, digital radiography, image quality, positioning accuracy

## Abstract

**Background:**

Image‐guided particle therapy (IGPT) has significantly advanced in recent years, particularly in the context of proton therapy. However, imaging solutions for carbon‐ion radiotherapy (C‐ion RT) remain limited.

**Purpose:**

This study introduces sliding‐gantry cone‐beam computed tomography (CBCT) and dual‐panel digital radiography (DR) systems, both mechanically independent of carbon‐ion delivery nozzles. We aim to evaluate the image quality metrics and verify the positioning accuracy of the imaging systems.

**Methods:**

Image quality was evaluated in terms of spatial resolution, low contrast resolution, image uniformity, and effective imaging area using a multi‐purpose imaging phantom, Catphan 700 phantom, and ImageJ software. The influences of planning computed tomography (CT) slice thicknesses (1–5 mm), radiation quality settings (90–130 kV), and registration algorithms (bony, grayscale, and fiducial marker registrations) on positioning accuracy were assessed using anthropomorphic head‐neck and thoracoabdominal phantom images. The clinical feasibility of both systems was validated in 22 enrolled patients.

**Results:**

The CBCT exhibited a lower in‐plane spatial resolution (2.50 line pairs per millimeter (lp/mm)) than DR (2.80 lp/mm). Spatial resolution of the CBCT system was measured at 0.90 lp/mm using the CTP 714 module of the Catphan 700 phantom. Both systems achieved a low contrast resolution of 2.30%. DR provided superior image uniformity (1.12%–1.40%) compared with CBCT (2.20%). The effective imaging areas were comparable between the CBCT and DR systems (99.30%–99.50%). Positioning accuracy varied with planning CT slice thicknesses, radiation quality settings, and registration algorithms, showing mean translation displacements ranging from 0.01 to 0.48 mm. CBCT achieved inter‐fraction translational positioning errors within 2 mm in 42.3% (22/52) of fractions and rotational positioning errors within 2° in 80.8% (42/52) of fractions, and DR achieved 33.8% (24/71) and 73.2% (52/71), respectively.

**Conclusion:**

The developed CBCT and DR systems achieved superior image quality and sub‐0.5 mm positioning accuracy. These findings support the clinical feasibility of integrating CBCT and DR imaging systems into the C‐ion RT workflow.

## 1 INTRODUCTION

Image‐guided particle therapy (IGPT) systems are being progressively adopted in clinical practice to improve target localization accuracy and therapeutic efficacy.^1^, ^2^ Modern proton therapy commonly utilizes orthogonal digital radiography (DR) combined with cone‐beam computed tomography (CBCT) for patient setup correction.^1^, ^2^, ^3^, ^4^, ^5^, ^6^ By contrast, the commercial solutions and clinical adoption of image guidance systems for carbon‐ion radiotherapy (C‐ion RT) remains limited because of the technology and implementation challenges as well as the limited number of operational C‐ion RT facilities.^7^ The variable relative biological effectiveness (RBE) distributions and higher biological effectiveness in C‐ion RT necessitate more sophisticated imaging techniques for accurate guidance and monitoring of treatments.^7^ Although CBCT is essential for IGPT to improve tumor localization accuracy,^2^, ^8^ C‐ion RT centers equipped with dedicated CBCT systems are rare in clinical practice.^4^ Currently, clinically operational C‐ion RT centers primarily utilize orthogonal X‐ray images combined with conventional in‐room computed tomography (CT) for patient setup verification.^9^ In addition to hardware challenges, the scarcity of software solutions is a typical constraint in modern imaging for IGPT clinical applications.^4^

The introduction of new IGPT systems in clinical implementation involves acceptance testing and commissioning. This process necessitates benchmarking of image quality metrics, including spatial resolution, low contrast resolution, image uniformity, and effective imaging area.^10^, ^11^ In image‐guided C‐ion RT, incorporating these metrics into benchmarking is essential for validating new imaging system, thereby ensuring that they fulfill the clinical performance standards of C‐ion RT. The accuracy standards in particle therapy should consider particle‐specific characteristics, thereby diverging from photon image‐guided radiotherapy (IGRT) practices.^4^ Proton therapy typically requires patient alignment within 1‐mm accuracy comparable to that of photon radiosurgery.^1^ By contrast, compared with the use of protons, the use of carbon ions in radiotherapy is distinguished by higher RBE in the Bragg peak region and steeper dose gradients, rendering positional errors more impactful on the delivered biological dose.^12^ Consequently, C‐ion RT may require a tighter positioning accuracy threshold, such as ≤0.5 mm, than proton therapy.^8^ Evaluating image guidance accuracy involves quantifying the influences of factors such as planning CT slice thickness, radiation quality settings, and registration algorithms on positioning accuracy. Phantom studies have demonstrated that slice thickness affects positioning accuracy, underscoring the need for careful selection of imaging parameters.^13^ Optimizing radiation quality, specifically X‐ray tube voltage (kV), is crucial for balancing the radiation dose with registration accuracy. Several studies have revealed the importance of adjusting kV to achieve this balance.^14^, ^15^ Furthermore, positioning accuracy depends on the registration method, particularly in two‐dimensional (2D) to 3D registration, where algorithmic choices influence setup error detection and correction.^16^ Evaluating the effects of these factors on positioning accuracy is critical for establishing reproducible clinical protocols for precision carbon‐ion therapy.

The integration of gantry‐mounted IGPT systems into C‐ion RT poses significant challenges in terms of technical characteristics and financial costs, particularly for fixed‐beam port systems. The development of an independent IGPT system could be a strategic approach to address these limitations. In this study, both dedicated sliding‐gantry CBCT and dual‐panel DR systems, mechanically independent of fixed carbon‐ion beamlines, were developed and clinically implemented for image‐guided C‐ion RT. Image acquisition and registration were performed using in‐house Radsnipe Image Analysis software (version 5.3.0; Shinetone Medical Equipment Co., Ltd., China). This paper reports the acceptance and commissioning results of image quality and system positioning accuracy as follows: First, image quality metrics, including spatial resolution, low contrast resolution, image uniformity, and effective imaging area, were evaluated. Next, positioning accuracy related to planning CT slice thickness, radiation quality settings, and registration algorithm was assessed. Finally, the target positioning accuracy for opposing dual‐field irradiation was investigated by comparing residual errors in phantom positioning between 0° and 180° couch rotations, and clinical feasibility was demonstrated in an initial patient cohort. Congruence testing of imaging and treatment isocenters, device specifications, and methodological procedures are documented in the Supplementary Materials (Figure S1, Tables S1–S4).

## 2 MATERIAL AND METHODS

### 2.1 Imaging devices and system framework

**FIGURE 1:**
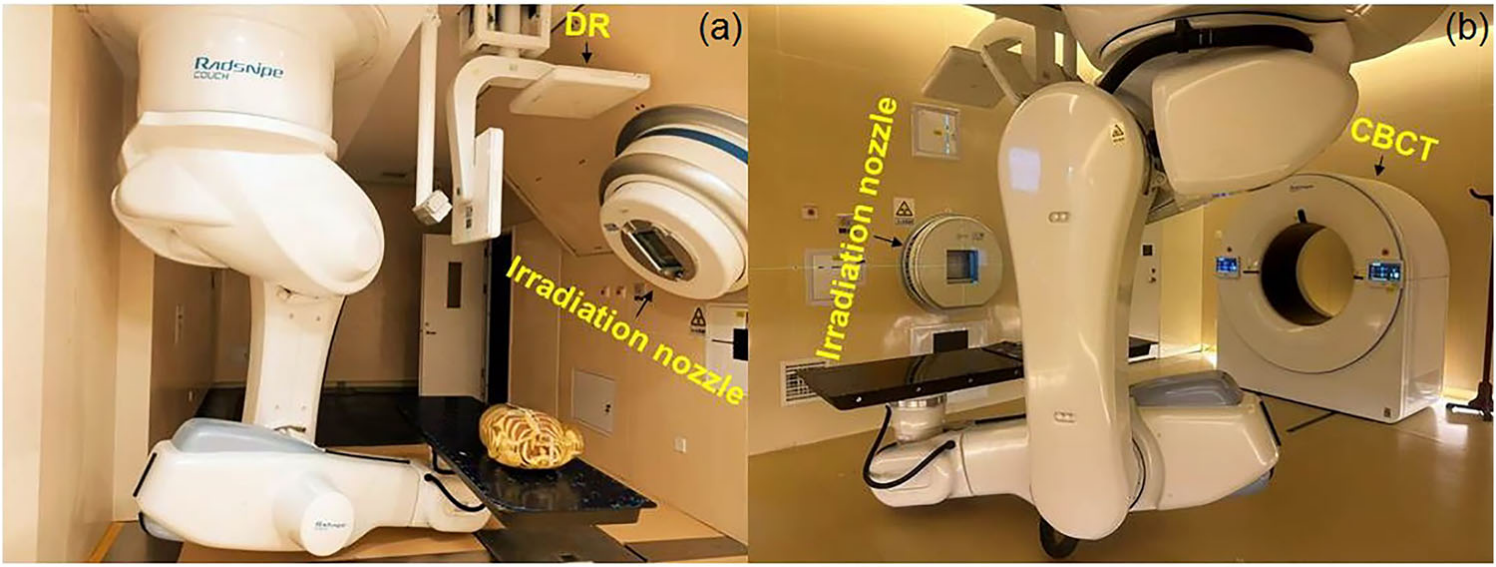
Photographs showing the image‐guided carbon‐ion therapy system configuration. (a) Digital radiography used for the 45° uniform scanning beamline. (b) CBCT employed in the horizontal pencil beam scanning beamline.

In Heavy Ion Research Facility in Lanzhou (HIRFL, Figure S2), the CBCT and DR systems (Shinetone Medical Equipment Co., Ltd., China) were mechanically independent of the fixed carbon‐ion port systems, as shown in Figure 1. The sliding‐gantry CBCT rotates from –120° to +120° to capture 240 images using full‐fan mode.

**FIGURE 2:**
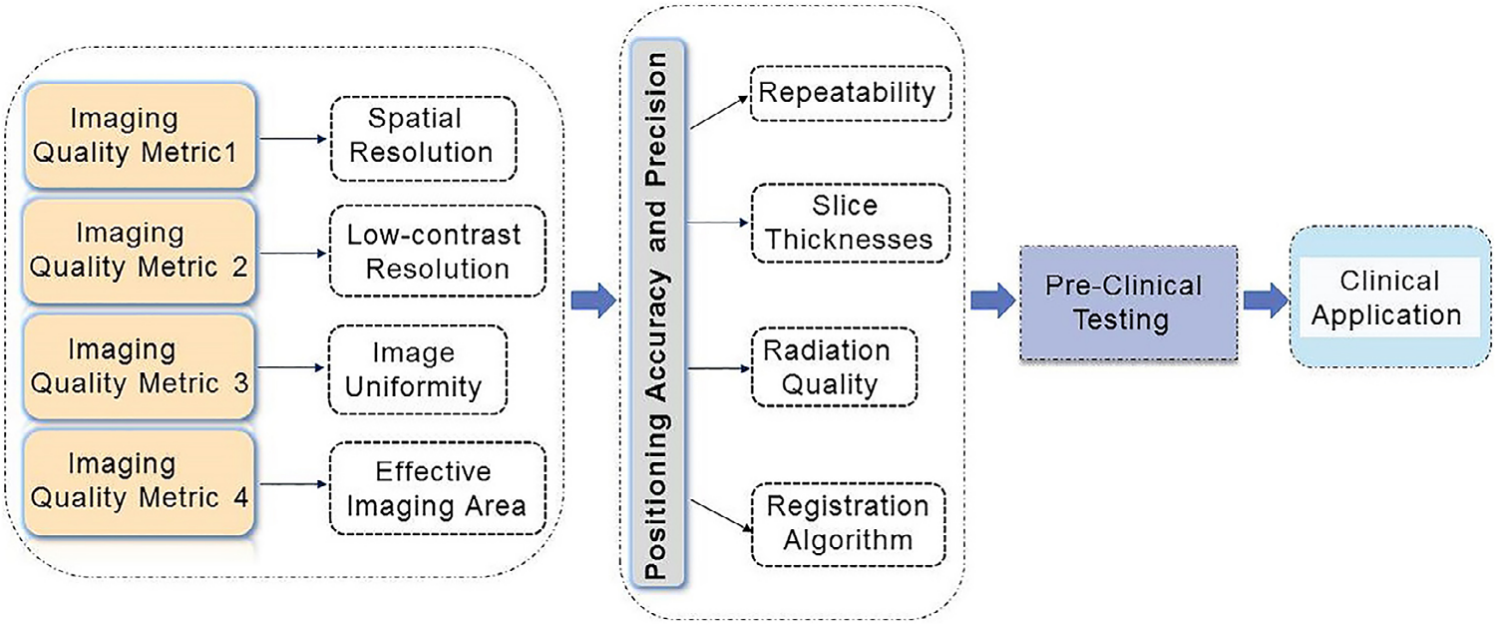
Overview of the study workflow.

Figure 2 schematically illustrates the experimental framework and outlines four fundamental steps described in subsequent subsections.

### 2.2 Imaging quality metrics

#### 2.2.1 Spatial resolution and low contrast resolution

**FIGURE 3:**
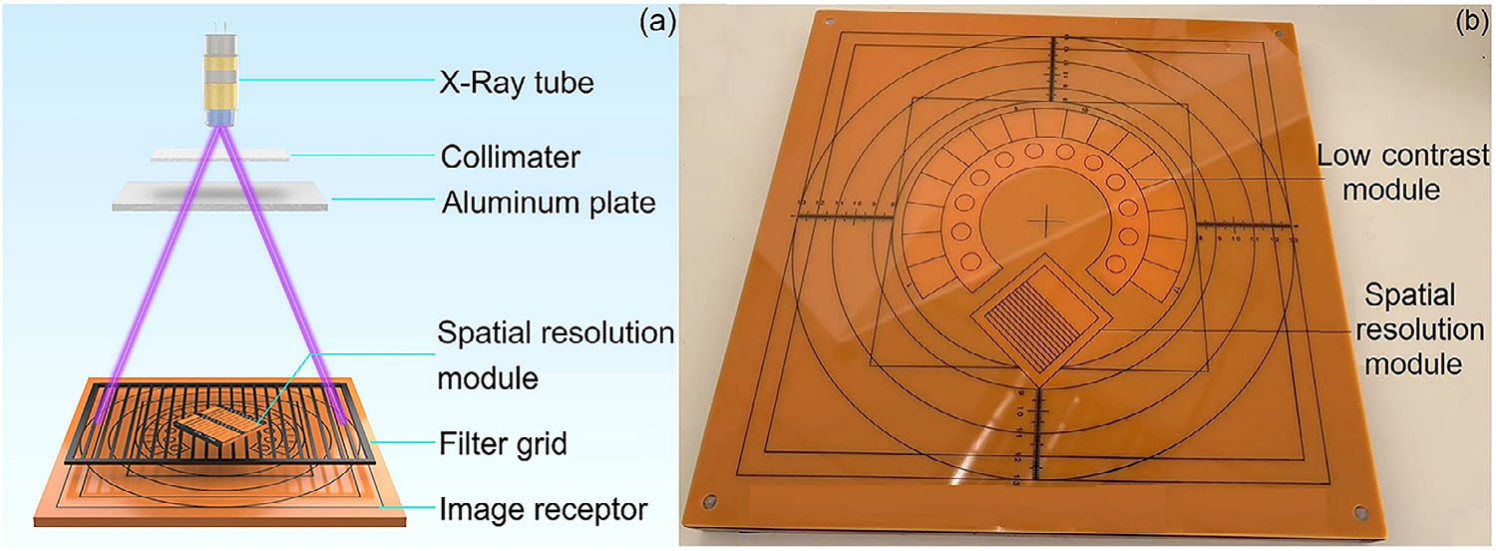
Image acquisition for spatial resolution evaluation. (a) A schematic drawing the main components for spatial resolution measurement. (b) Front view of multi‐purpose imaging phantom with spatial resolution and low contrast modules.

A multi‐purpose imaging phantom (Xinhuiren Imaging Technology Co., Ltd., China) and ImageJ software (version 1.49; National Institutes of Health, USA, http://imagej.nih.gov/ij/) were used to evaluate the in‐plane spatial resolution of CBCT and DR images. As illustrated in (Figure 3a,b), the spatial resolution module of the phantom was positioned at a 45° angle relative to the anti‐scattering filter grid, with a 20‐mm‐thick aluminum plate centered on the beam axis serving as an attenuator. Low contrast resolution was quantified by calculating the contrast‐to‐noise ratio (CNR) without the aluminum attenuator. An air kerma detector and the low contrast module of the multi‐purpose imaging phantom were used for measurement, as shown in Figure 3b. For the CBCT system, spatial resolution was further assessed by scanning the CTP 714 high resolution module of the Catphan 700 phantom (The Phantom Laboratory, Salem, NY, USA) and analyzing line‐pair patterns. Additional details are provided in Figure S3 and Table S5.

**FIGURE 4:**
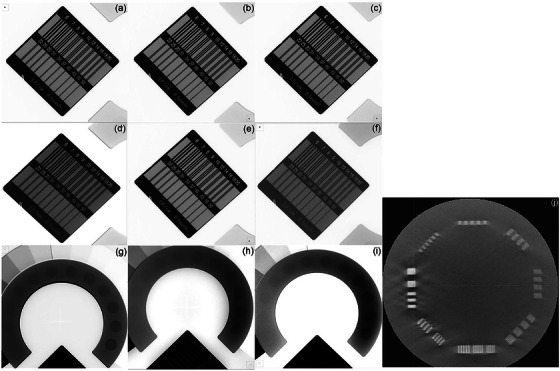
Radiographic images of spatial resolution and low contrast resolution acquired using multi‐purpose imaging phantom and Catphan 700 phantom. Unattenuated in‐plane spatial resolution images for (a) CBCT, (b) DR1, and (c) DR2. Attenuated spatial resolution images for (d) CBCT, (e) DR1, and (f) DR2. Low contrast resolution images for (g) CBCT, (h) DR1, and (i) DR2. (j) Image of CTP 714 module of the Catphan 700 phantom. DR1 and DR2 are the flat panel detectors of the dual‐panel DR system. DR, digital radiography; CBCT, cone‐beam computed tomography.

Figure 4a–c illustrate the unattenuated in‐plane spatial resolution images, and Figure 4d–f display the corresponding attenuated images. Low contrast resolution images are displayed in Figure 4g–i. Figure 4j shows slices of the CTP 714 module of the Catphan 700 phantom acquired with the CBCT system. The technical parameters and modulation transfer function characteristics were validated by repeated measurements to ensure that the imaging systems deliver consistent and reliable results.

#### 2.2.2 Image uniformity

**FIGURE 5:**
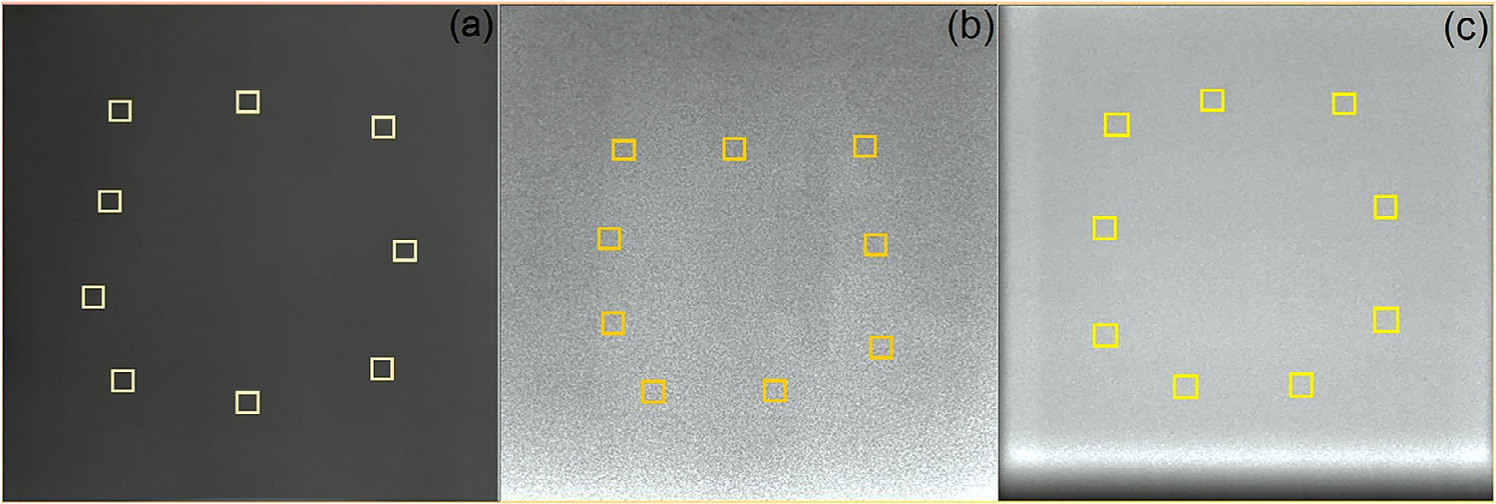
Images for uniformity assessment. Representative uniformity images of (a) CBCT, (b) DR1, and (c) DR2. DR1 and DR2 are the flat panel detectors of the dual‐panel DR system.

Abbreviations: ROI, region of interest; DR, digital radiography; CBCT, cone‐beam computed tomography.

As shown in Figure 5, nine square regions of interest (ROIs), each encompassing approximately 1% of the total area of the detector, were distributed peripherally, ensuring a minimum distance of twice the ROI side length between the ROI boundary and detector edge. Using ImageJ software, a 64 × 64 pixel subregion was extracted from each ROI, and the mean pixel intensity V_i_ was computed for each ROI. Image uniformity was quantified as the ratio of the standard deviation R to the mean pixel intensity V_m_ given by

(1) $$\mathrm{Uniformity}\;=\frac{R}{V_{m}}=\frac{\sqrt{\frac{1}{8}\sum_{i=1}^{9}{\left(V_{i}-V_{m}\right)}^{2}}}{\frac{1}{9}\sum_{i=1}^{9}V_{i}}\times 100\%\;$$

#### 2.2.3 Effective imaging area

**FIGURE 6:**
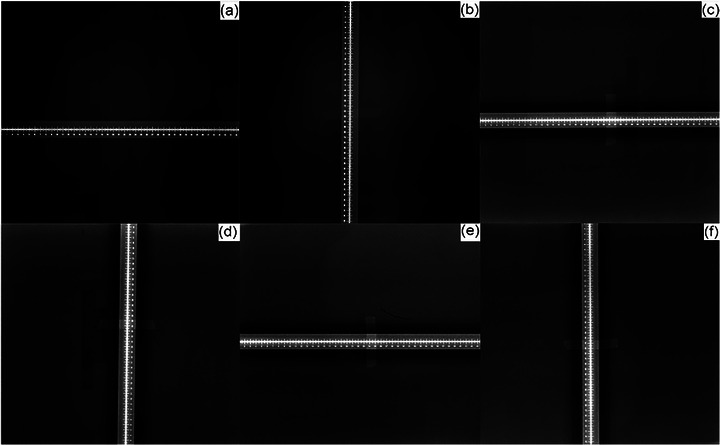
Radiographic images of effective imaging area. Radiopaque ruler measurements for CBCT image in (a) x‐axis and (b) y‐axis; radiopaque ruler measurements for DR1 image in (c) x‐axis and (d) y‐axis; radiopaque ruler measurements for DR2 image in (e) x‐axis and (f) y‐axis. DR1 and DR2 are the flat panel detectors of the dual‐panel DR system. DR, digital radiography; CBCT, cone‐beam computed tomography.

After removing the filter grid (Figure 3a), a radiopaque ruler was positioned sequentially along the x‐ and y‐axes of the flat panel detector to acquire radiographic images. As shown in Figure 6, the spacing between the scale markings was measured using ImageJ to obtain the physical dimensions x__measured_ (mm) and y__measured_ (mm). The effective imaging areas along both axes can be expressed as

(2) $$\;\mathrm{Effective}\;\mathrm{imaging}\;\mathrm{are}a_{x}=\frac{x_{\_\mathrm{measured}}}{x_{\_\mathrm{specified}}}\times 100\%\;$$

(3) $$\;\mathrm{Effective}\;\mathrm{imaging}\;\mathrm{are}a_{y}=\frac{y_{\_\mathrm{measured}}}{y_{\_\mathrm{specified}}}\times 100\%\;$$

The nominal imaging area is 427 × 427 mm^2^ (x__specified _= y__specified _= 427 mm).

### 2.3 Evaluation on positioning accuracy

Positioning accuracy was evaluated using anthropomorphic head‐neck and thoracoabdominal phantoms (Chengdu Dosimetric Phantom, CPET Co. Ltd, China). Initially, the reference isocenter in the phantom was aligned with the room isocenter, and the target volume within the phantom was positioned at the reference point F__reference_ (0, 0, 0). CT images acquired at this position served as the target images. The phantom was then displaced along the negative x (lateral), y (longitudinal), and z (vertical) axes to the actual coordinate point F__offset_ (x__0_, y__0_, z__0_), with x__0 _= y__0 _= z__0_ = –10 mm for the head‐neck phantom and –15 mm for the thoracoabdominal phantom, denoted as the actual target shifts. CBCT and DR images captured at the F__offset_ coordinate point were used as source images for subsequent registration using in‐house Radsnipe Image Analysis software. To validate the computational accuracy of the software, 10 repeated CBCT and 10 repeated DR scans obtained at coordinate point F__offset_ were aligned with the target images (slice thickness of 1 mm) using grayscale registration (Figure S4–6). The translation displacement (TD) of the calculated coordinate point F__cal_ and actual coordinate point F__offset_ were quantified using Equation (4).

(4) $$\mathrm{TD}\;=\sqrt{\Delta x^{2}+\Delta y^{2}+\Delta z^{2}}\;\;$$

where ∆x, ∆y, and ∆z represent the differences in coordinates between two points along the x‐, y‐, and z‐ axes, respectively.

To assess the impact of planning CT slice thickness on positioning accuracy (Figure S7), source images were registered with target images acquired using 1, 3 (baseline), and 5‐mm slice thicknesses to obtain the registered coordinate points F__1 mm_ (*x*__1 mm_, *y*__1 mm_, *z*__1 mm_), F__3 mm_ (*x*__3 mm_, *y*__3 mm_, *z*__3 mm_), and F__5 mm_ (*x*__5 mm_, *y*__5 mm_, *z*__5 mm_). The translation displacements of F__1 mm_ versus F__3 mm_ and F__5 mm_ versus F__3 mm_ were calculated using Equation (4). The impact of radiation quality on positioning accuracy was evaluated by registering the source images acquired using the minimum, baseline, and maximum tube voltages with the target images to obtain the coordinate points F__min_ (*x*__min_, *y*__min_, *z*__min_), F__baseline_ (*x*__baseline_, *y*__baseline_, *z*__baseline_), and F__max_ (*x*__max_, *y*__max_, *z*__max_). The translation displacements between F__min_ versus F__baseline_ and F__max_ versus F__baseline_ were derived using Equation (4). The influence of the registration algorithms on positioning accuracy was evaluated by aligning the source images with the target images using bony‐based, grayscale‐based (baseline), and fiducial marker‐based registrations to obtain the coordinate points F__ bone_ (*x*__bone_, *y*__bone_, *z*__bone_), F__ gray_ (*x*__ gray_, *y*__ gray_, *z*__ gray_), and F__marker_ (*x*__marker_, *y*__marker_, *z*__marker_) (Figure S8). The translation displacements of F__bone_ versus F__gray_ and F__marker_ versus F__gray_ were calculated using Equation (4). Each of the three experimental procedures was independently repeated three times, with results expressed as means. All displacements were within the 0.5‐mm threshold.

### 2.4 Pre‐clinical testing and clinical application

**FIGURE 7:**
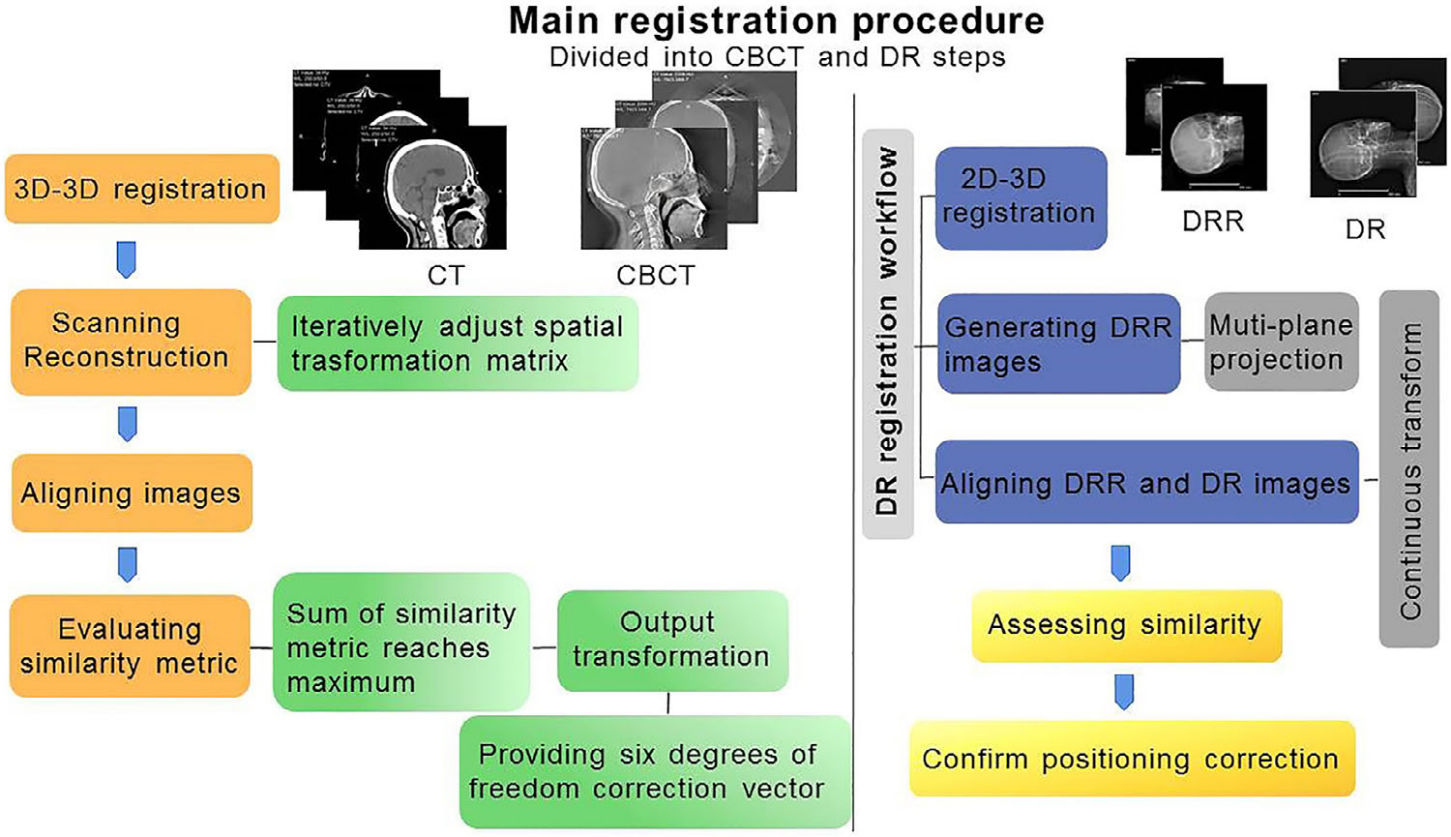
Schematic representation illustrating the registration framework of the CBCT and DR systems. The registration process employs iterative optimization to refine transformation parameters until alignment metrics are maximized. Algorithm convergence ensures subvoxel geometric correspondence. DR, digital radiography; DRR, digitally reconstructed radiographs; CBCT, cone‐beam computed tomography; 2D, two‐dimensional; 3D, three‐dimensional.

Preclinical testing refers to evaluating the target positioning accuracy for opposing dual‐field irradiation in carbon ion therapy. Following the recommendations of the American Association of Physicists in Medicine (AAPM) Task Group (TG) 179,^17^ the positioning accuracy was evaluated by comparing the post‐correction residual errors between the 0° and 180° couch positions using an anthropomorphic phantom. The procedure was repeated five times, and the results are presented as absolute residual errors. Based on a 95% confidence interval (CI),^17^ the residual error should be within 0.5 mm. Inter‐fractional patient positioning errors were assessed for 22 participants enrolled in a carbon‐ion clinical trial at HIRFL between October 2022 and January 2023. A total of 52 CBCT scans and 71 DR images were acquired and analyzed (Figure S9,10). Figure 7 shows the registration workflow. Spatial transformations were optimized by maximizing the similarity metric between the stationary source image and the moving target image. Setup errors were quantified as six‐degree‐of‐freedom (6DoF) vectors, including translations (lateral, longitudinal, and vertical) and rotations (pitch, roll, and yaw).

### 2.5 Statistical analysis

Statistical analyses were performed using the GraphPad Prism software (version 10.5; La Jolla, CA, USA). Each image quality metric was assessed through three independent repeated acquisitions under identical conditions, and the results were presented as means. Residual errors are expressed as means ± standard deviation (SD) with 95% CI.

## 3 RESULTS

### 3.1 Image quality metrics

Table 1 presents the image quality metrics of the CBCT and DR systems. DR achieved an in‐plane spatial resolution of 2.80 lp/mm, exceeding the 2.50 lp/mm of CBCT. The spatial resolution of CBCT was measured at 0.90 lp/mm using the CTP 714 module of the Catphan 700 phantom. The low contrast resolution was equivalent for both systems at 2.30%. The DR system demonstrated superior uniformity (1.12%–1.40%) compared with CBCT (2.20%). Both systems achieved excellent effective imaging areas (DR, 99.50%; CBCT, 99.30%).

**TABLE 1:** Image quality metrics of CBCT and DR systems (means).

Metrics	Protocols	Parameters	Means
In‐plane spatial resolution	Multi‐purpose imaging phantom No attenuator	68 kV, 20 mA, 1 mAs (CBCT)	2.50 lp/mm
		68 kV, 80 mA, 1 mAs (DR1) 68 kV, 80 mA, 5 mAs (DR2)	2.80 lp/mm 2.80 lp/mm
	Multi‐purpose imaging phantom Aluminum attenuator	82 kV, 80 mA, 4 mAs (CBCT)	2.50 lp/mm
		80 kV, 100 mA, 10 mAs (DR1) 80 kV, 100 mA, 10 mAs (DR2)	2.80 lp/mm 2.80 lp/mm
Low contrast resolution	Multi‐purpose imaging phantom No attenuator	75 kV, 80 mA, 8 mAs (CBCT)	2.30 %
		82 kV, 250 mA, 25 mAs (DR1; DR2)	2.30%
Spatial resolution	Catphan 700 phantom	80 kV, 40 mA, 50 mAs (CBCT)	0.90 lp/mm
Image uniformity	Aluminum attenuator	68 kV, 10 mA, 5 mAs (CBCT)	2.20%
		68 kV, 10 mA, 1 mAs (DR1) 68 kV, 50 mA, 5 mAs (DR2)	1.12% 1.40%
Effective imaging region	68 kV, 10 mA, 1 mAs (CBCT); 50 kV, 50 mA, 2.5 mAs (DR1); 68 kV, 50 mA, 2.5 mAs (DR2)		
Lateral		x__measured_ = x_2−_x_1 _= 425−1 = 424 mm (CBCT)	99.30%
		x__measured_ = x_2−_x_1 _= 435−10 = 425 mm (DR1, DR2)	99.50%
Longitudinal		y__measured_ = y_2−_y_1 _= 434−10 = 424 mm (CBCT)	99.30%
		y__measured_ = y_2−_y_1 _= 436−11 = 425 mm (DR1, DR2)	99.50%

*Note*: x_1_, x_2_, y_1_, and y_2_ denote the coordinates of the radiopaque ruler along the x‐ and y‐axes, as measured in the radiographic image. DR1 and DR2 are the flat panel detectors of the dual‐panel DR system.

Abbreviations: CBCT, cone‐beam computed tomography; DR, digital radiography; lp/mm, line pairs per millimeter.

### 3.2 Evaluation on positioning accuracy

**FIGURE 8:**
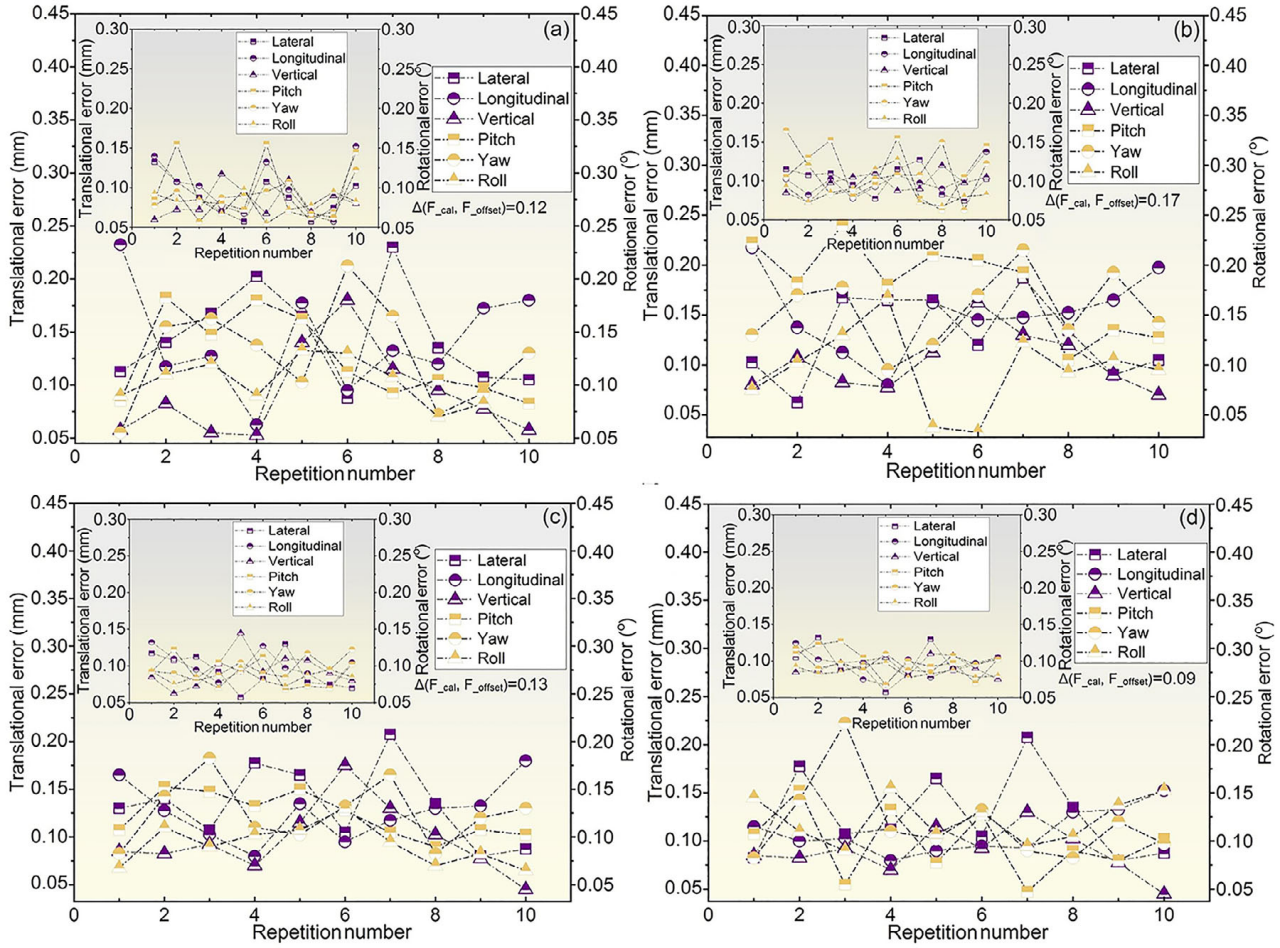
Comparison of positioning errors for 10 repetitions at a fixed target. Translational and rotational positioning errors obtained from (a) DR__guided head‐neck phantom_, (b) DR__guided thoracoabdominal phantom_, (c) CBCT__guided head‐neck phantom_, and (d) CBCT__guided thoracoabdominal phantom_. Insets depict the post‐correction residual errors. Δ(F__cal_, F__offset_) denotes translation displacements between the calculated and actual target positions by Equation (4). DR, digital radiography; CBCT, cone‐beam computed tomography.

As shown in Figure 8, the differences between the calculated and actual shifts in 10 repetitions were all less than 0.3 mm and 0.3°, with mean translation displacements of 0.12 mm (DR__guided head‐neck phantom_), 0.17 mm (DR__guided thoracoabdominal phantom_), 0.13 mm (CBCT__guided head‐neck phantom_), and 0.09 mm (CBCT__guided thoracoabdominal phantom_). Following positioning errors correction, residual errors acquired by the Radsnipe software were below 0.15 mm in translation and below 0.17° in rotation in all cases, as illustrated in the small insets.

As presented in Table 2, 3, 4, translation displacements ranged from 0.01 to 0.48 mm under varying planning CT slice thicknesses, radiation quality settings, and registration algorithms.

**TABLE 2:** Positioning accuracies for various planning CT slice thicknesses (means, mm).

	F__1 mm_			F__5_ _mm_			F__3 mm_			Translation displacements	
Protocols	x__1_ _mm_	y__1_ _mm_	z__1_ _mm_	x__5_ _mm_	y__5_ _mm_	z__5_ _mm_	x__3_ _mm_	y__3_ _mm_	z __3 mm_	Δ(F__1 mm_, F__3 mm)_	Δ(F__5 mm_, F__3 mm_)
DR_HN_	−9.81	−9.95	−9.92	−9.77	−9.72	−9.85	−9.95	−10.16	−9.91	0.25	0.48
CBCT_HN_	−10.47	−9.76	−10.07	−10.55	−9.63	−9.85	−10.51	−9.55	−9.85	0.31	0.09
DR_T_	−15.15	−14.93	−14.92	−15.09	−15.09	−14.75	−15.23	−14.89	−14.51	0.42	0.34
CBCT_T_	−15.00	−15.04	−14.91	−15.04	−14.94	−14.90	−15.14	−14.93	−14.84	0.19	0.12

*Note*: The actual physical shifts were −10 mm for the head‐neck phantom and −15 mm for the thoracoabdominal phantom along the negative x‐, y‐, and z‐axes. Coordinate points F__1 mm_, F__5 mm_, and F__3 mm_ were calculated by registering the source images with target images acquired using slice thicknesses of 1, 5, and 3 mm, respectively. Δ(F__1 mm_, F__3 mm)_ and Δ(F__5 mm_, F__3 mm_) denote the translation displacements of F__1 mm_ versus F__3 mm_ and F__5 mm_ versus F__3 mm_ calculated using Equation (4).

Abbreviations: CBCT, cone‐beam computed tomography; CT, computed tomography; DR, digital radiography; HN, head‐neck; T, thoracoabdominal.

**TABLE 3:** Positioning accuracies for various radiation quality settings (means, mm).

	F__min_			F__max_			F__baseline_			Translation displacements	
Protocols	x__min_	y__min_	z__min_	x__max_	y__max_	z__max_	x__baseline_	y__baseline_	z__baseline_	Δ(F__min_, F__baseline)_	Δ(F__max_, F__baseline_)
DR_HN_	−9.80	−10.00	−9.89	−9.75	−9.98	−9.88	−9.81	−10.00	−9.89	0.01	0.06
CBCT_HN_	−10.28	−9.81	−10.27	−10.26	−9.82	−10.36	−10.26	−9.77	−10.30	0.05	0.08
DR_T_	−15.02	−15.00	−15.02	−15.01	−15.00	−15.02	−15.01	−15.00	−15.00	0.02	0.02
CBCT_T_	−14.83	−15.32	−15.03	−14.82	−15.31	−14.92	−14.92	−15.25	−14.94	0.15	0.12

*Note*: The actual physical shifts were −10 mm for the head‐neck phantom and −15 mm for the thoracoabdominal phantom along the negative x‐, y‐, and z‐axes. Coordinate points F__min_, F__baseline_, and F__max_ were calculated by registering the source images acquired using the minimum, baseline, and maximum tube voltages with the target images, respectively. Δ(F__min_, F__baseline)_ and Δ(F__max_, F__baseline_) denote translation displacements of F__min_ versus F__baseline_ and F__max_ versus F__baseline_ calculated using Equation (4).

Tube voltages for head‐neck phantom are 110 kV, 120 kV, and 130 kV for DR, and 90 kV, 100 kV, and 110 kV for CBCT. For the thoracoabdominal phantom, both DR and CBCT were operated at 110 kV, 120 kV, and 130 kV.

Abbreviations: CBCT, cone‐beam computed tomography; DR, digital radiography; HN, head‐neck; T, thoracoabdominal.

**TABLE 4:** Positioning accuracies for various registration algorithms (means, mm).

	F__ bone_			F__ marker_			F__ gray_			Translation displacements	
Protocols	x__bone_	y__bone_	z__bone_	x__ marker_	y__ marker_	z__ marker_	x__ gray_	y__ gray_	z__ gray_	Δ(F__bone_,F__gray_)	Δ(F__marker_, F__gray_)
DR_HN_	−10.12	−10.13	−10.08	−9.88	−9.76	−10.13	−10.08	−10.00	−10.02	0.21	0.33
CBCT_HN_	−10.08	−9.89	−10.06	−10.25	−10.09	−9.79	−10.13	−10.06	−10.09	0.20	0.34
DR_T_	−15.11	−14.93	−15.24	−15.04	−15.09	−15.07	−15.15	−14.93	−14.92	0.32	0.25
CBCT_T_	−15.18	−14.90	−14.99	−14.91	−15.13	−14.86	−14.88	−15.00	−14.98	0.32	0.20

*Note*: The actual physical shifts were −10 mm for the head‐neck phantom and −15 mm for the thoracoabdominal phantom along the negative x‐, y‐, and z‐axes. Coordinate points F__bone_, F__gray_, and F__ marker_ were calculated by registering the source images with target images using bone, gray (baseline), and marker registrations, respectively. Δ(F__bone_, F__gray_) and Δ(F__marker_, F__gray_) denote translation displacements of F__bone_ versus F_0, gray_ and F__marker_ versus F__gray_ calculated using Equation (4).

Abbreviations: CBCT, cone‐beam computed tomography; DR, digital radiography; HN, head‐neck; T, thoracoabdominal.

### 3.3 Pre‐clinical testing

At a 0° couch angle (Table 5), the mean residual errors were below 0.48 mm in translation and below 0.72° in rotation. For 180° (Table 6), mean residual errors reduced to less than 0.36 mm in translation and less than 0.2° in rotation.

**TABLE 5:** Translational and rotational residual errors at couch 0° (means ± SD; 95% CI).

	Translational residual errors (mm)			Rotational residual errors (°)		
Protocols	x	y	z	pitch	roll	yaw
Head‐neck phantom	0.41 ± 0.19	0.15 ± 0.11	0.32 ± 0.18	0.72 ± 0.27	0.45 ± 0.22	0.36 ± 0.13
	(0.34, 0.49)	(0.12, 0.25)	(0.26, 0.41)	(0.62, 0.79)	(0.33, 0.55)	(0.33, 0.44)
Thoracoabdominal phantom	0.42 ± 0.21	0.26 ± 0.13	0.48 ± 0.25	0.66 ± 0.24	0.20 ± 0.08	0.19 ± 0.06
	(0.35, 0.51)	(0.20, 0.39)	(0.34, 0.59)	(0.52, 0.71)	(0.23, 0.42)	(0.13, 0.31)

Abbreviations: CI, confidence interval. The reported means and standard deviation were calculated based on the absolute residual errors; SD, standard deviation.

**TABLE 6:** Translational and rotational residual errors at couch 180° (means ± SD; 95% CI).

	Translational residual errors (mm)			Rotational residual errors (°)		
Protocols	x	y	z	pitch	roll	yaw
Head‐neck phantom	0.33 ± 0.15	0.23 ± 0.14	0.36 ± 0.14	0.11 ± 0.06	0.04 ± 0.01	0.17 ± 0.08
	(0.23, 0.42)	(0.19, 0.38)	(0.26, 0.46)	(0.09, 0.18)	(0.02, 0.09)	(0.13, 0.28)
Thoracoabdominal phantom	0.27 ± 0.15	0.32 ± 0.19	0.18 ± 0.13	0.18 ± 0.12	0.07 ± 0.02	0.16 ± 0.05
	(0.21, 0.49)	(0.26, 0.39)	(0.14, 0.32)	(0.12, 0.29)	(0.04, 0.12)	(0.13, 0.31)

Abbreviations: CI, confidence interval. The reported means and standard deviation were calculated based on the absolute residual errors; SD, standard deviation.

### 3.4 Clinical application

**FIGURE 9:**
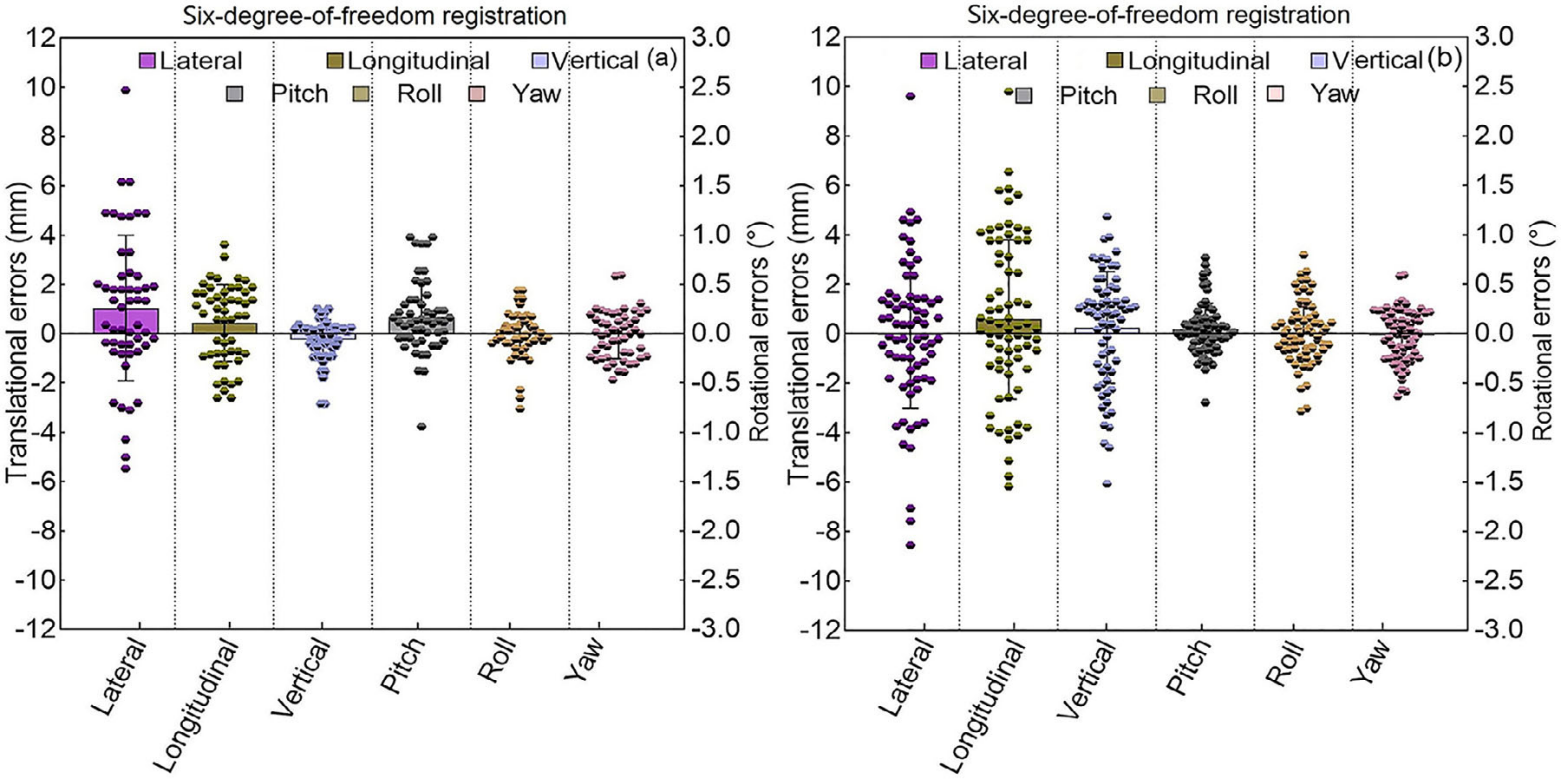
Inter‐fraction patient positioning errors acquired by registering DR (a) and CBCT (b) images to patient planning CT image. Abbreviations: CBCT, cone‐beam computed tomography; DR, digital radiography; CT, computed tomography.

Figure 9 shows the daily patient positioning errors of the DR and CBCT systems. DR exhibited translational positioning error less than 9.8 mm, with 33.8% of fractions (24/71) within 2 mm. The maximum rotational positioning errors were 3.08° in pitch, 3.2° in roll, and 2.5° in yaw, and 73.2% of fractions (52/71) were within 2°. CBCT achieved a translational positioning error within 2 mm in 42.3% of the fractions (22/52), with a maximum error of 9.55 mm. For the rotational positioning errors, CBCT achieved 80.8% of the fractions (42/52) below 2° and maxima of 2.86° (pitch), 3.92° (roll), and 3.05° (yaw).

## 4 DISCUSSION

Analogous to onboard IGRT systems, the in‐house developed DR and CBCT imaging systems provided 2D planar radiographs and 3D volumetric images for patient setup corrections. The results show that the CBCT system achieved an in‐plane spatial resolution of 2.50 lp/mm. Spatial resolution of the CBCT system was measured at 0.90 lp/mm using the CTP 714 module of the Catphan 700 phantom, superior to the established proton CBCT systems.^1^, ^12^ Both systems achieved an identical low contrast resolution of 2.30%, outperforming tomotherapy system, but remaining inferior to the gantry‐mounted CBCT systems.^18^ Image uniformity, defined as the consistency of pixel intensity across the field of view,^19^ was 2.2% for CBCT and ranged from 1.12% to1.4% for DR, which was superior to the Elekta XVI system.^20^ X‐ray imaging frequently generates non‐diagnostic areas, primarily caused by image artifacts, incomplete data acquisition (such as truncated projections in CBCT), and detector limitations. Accurately identifying the effective imaging area is critical for optimizing storage compression, computational load, and image processing efficiency, particularly for data‐intensive volumetric imaging.^20^ Quantitative analysis revealed effective imaging areas of 99.30% for CBCT and 99.50% for DR. Each CBCT scan acquires 240 individual projection images, and based on a maximum pixel count of 9,437,184 per image and a 16‐bit analog‐to‐digital conversion bit depth (Table S3), the total raw data volume per CBCT scan was estimated to be approximately 4320.00 megabyte (MB), while that of a single DR scan was approximately 18.00 MB. Notably, a higher effective imaging area corresponds to a lower proportion of redundant data in the acquired scans. Accordingly, redundant data were reduced to 30.20 MB per CBCT scan and 0.09 MB per DR scan. The high effective imaging areas facilitate the transmission of larger volumes of clinically relevant information within the same file sizes, while reducing image transfer latency. Additionally, the low redundancy data of both modalities enhances the efficiency of storage resource utilization. In the context of carbon‐ion therapy, these efficiency improvements contribute to faster CBCT reconstruction and patient alignment verification, therapy reducing setup time in the treatment suite and enabling clinics to optimize daily patient throughput. Collectively, the high effective imaging areas of CBCT and DR systems significantly reduce redundant data processing and improve the clinical workflow efficiency.

Repeatability tests were performed using anthropomorphic phantoms to validate the computational accuracy of the in‐house Radsnipe Image Analysis software. The mean translation displacement between the calculated and actual coordinate points for a fixed target was less than 0.17 mm, significantly within the 0.5‐mm clinical threshold (Figure 8). These results demonstrate the high computational precision and operational stability of the software, providing a basis for evaluating positioning accuracy in phantom studies and clinical practice. Planning CT slice thickness critically influences positioning accuracy because of partial volume effects and target delineation uncertainties. Thinner CT slices (≤ 1 mm) provide superior spatial resolution, facilitating the detection of subtle anatomical displacements and mitigating partial volume effects in soft tissue targets. However, compared with thicker slice scans, thinner slice scans generally impose severe wear on imaging hardware and require considerable storage space. Thicker CT slices (≥ 3 mm) reduce image noise, benefiting rigid alignment but may obscure fine anatomical details, potentially biasing setup error estimates.^21^, ^22^, ^23^ The study quantified the translation displacements for registrations using 1‐ and 5‐mm planning CT slices against registration using a 3‐mm reference slice thicknesses, with values ranging from 0.09 to 0.48 mm (Table 2). Although phantom studies demonstrated that 1‐mm CT slice thickness improved positioning accuracy over 3‐mm slice thickness, no significant difference was observed in the clinical setup precision between the two slice thicknesses.^12^ This shows that although thinner slices enhance accuracy in controlled settings, the clinical advantage may not be as pronounced. Our findings demonstrate that, compared with 3‐mm slices, 1‐ and 5‐mm slice thicknesses yield clinically negligible differences in registration accuracy. Therefore, a 3‐mm slice thickness is recommended as the clinical standard for planning CT in C‐ion RT, balancing CT equipment wear, storage space, treatment planning efficiency, and registration accuracy.

Radiation quality, primarily determined by tube voltage,^24^ may influence image quality metrics, including spatial resolution, low contrast resolution, and registration accuracy in certain scenarios. Low energy X‐rays (lower kV) enhance the image contrast between soft tissue and bone by amplifying photoelectric absorption, improving the differentiation of subtle anatomical boundaries. This capability facilitates the detection of fine positional deviations such as spinal registration.^25^ High kV imaging mitigates quantum noise and improves image uniformity by reducing attenuation differences between tissues,^5^ thereby enhancing the low contrast resolution and signal‐to‐noise ratio performances in homogeneous regions. This effect is advantageous for distinguishing tissues with minimal density differences. However, high‐kV imaging reduces the contrast between soft tissue and bone by suppressing photoelectric effects, mitigating differences in pixel intensity within the image, and potentially increasing the registration uncertainty for minor displacements.^25^ Quantitative analysis reveals that the maximum translation displacement between registrations performed at the lowest and highest kV settings and those performed at baseline kV is 0.15 mm, below the clinically accepted threshold of 0.5 mm (Table 3). Given the limited impact of kV variations on registration accuracy and the standardization of imaging protocols, 120 kV is recommended as the default setting for routine CBCT and DR imaging to prioritize radiation dose without compromising positioning accuracy, although pediatric and obese patients require adjusted kV settings.

The selection of an image registration algorithm has significant clinical implications, necessitating a balance between registration accuracy, computational efficiency, and patient‐specific factors. Bony registration offers high accuracy and computational speed for rigid anatomical structures, but fails to account for soft tissue deformation, limiting its utility for non‐osseous targets. Grayscale registration leverages global intensity information to enable robust multi‐modal fusion and to capture soft tissue displacement. However, susceptibility to noise artifacts, high computational burden, and prolonged runtime compromise clinical workflow efficiency. Fiducial marker matching provides high precision through direct tracking of implanted markers, with accuracy primarily determined by the spatial distribution of the markers. However, its invasive characteristics may introduce risks of complications, such as bleeding and infection. For a given imaging datasets, the difference in target registration accuracy using different registration algorithms is a key clinical consideration. Our findings indicate that the observed translation displacements ranged from 0.20 to 0.34 mm when comparing bone‐ and fiducial marker‐based methods with grayscale‐based method, below the 0.5‐mm tolerance threshold (Table 4). These inter‐algorithm differences in registration accuracy are clinically negligible, reflecting significant advancements in registration algorithms with enhanced procedural accuracy.^26^, ^27^ Accordingly, bone‐based rigid registration for rapid initial patient alignment and grayscale‐based deformable registration for target volume and organs at risk alignment were strategically selected to balance efficiency and accuracy.

Image registration is typically evaluated through physical phantom verification, digital phantom analysis, and clinical dataset validation.^17^ This study utilized anthropomorphic phantoms and clinical datasets to validate a dual‐beam C‐ion RT workflow. After rotating the couch from 0° to 180° and confirming laser‐isocenter alignment, CBCT and DR images acquired at the 180° position were registered with the planning CT image. The mean translational residual errors at 0° and 180°couch angles was within the range of 0.18–0.48 mm (Tables 5 and 6). These findings further demonstrate that the developed CBCT and DR systems meet the sub‐0.5 mm positioning accuracy requirements, maintaining the patient positioning accuracy essential for opposing dual‐field irradiation in carbon ion therapy. After validating sub‐0.5 mm positioning accuracy in phantom studies, we evaluated the initial clinical applications of CBCT and DR image guidance for C‐ion RT in 22 patients. The results indicated that CBCT provided superior translational positioning accuracy, and DR maintained rotational accuracy that was non‑inferior to CBCT (Figure 9). These findings support the technical and clinical feasibility of dual‐modality imaging guidance for C‐ion RT. The comparable rotational accuracy between CBCT (80.8% of fractions < 2°) and DR (71.8% of fractions < 2°) validates the reliability of both systems in mitigating rotational uncertainties (Figure 9). This is particularly important for carbon‐ion beams given their finite range and pronounced sensitivity to anatomical misalignment.

Finally, this study was limited to a relatively small patient cohort during clinical validation, which may compromise its capability to detect less frequent positional errors or system‐specific issues. Furthermore, as a characterization study, the results reflect system performance under controlled conditions and short‐term clinical use. Future studies will expand datasets by including larger and more diverse patient cohorts, along with more extensive image data. These efforts will improve the generalizability of the CBCT and DR systems while enabling long‐term performance monitoring and a more comprehensive assessment of the clinical utility.

## 5 CONCLUSION

This study introduces in‐house nozzle‐independent sliding‐gantry CBCT and dual‐panel DR imaging systems. The systems demonstrated high image quality and achieved sub‐0.5 mm positioning accuracy in phantom studies. These findings support the clinical feasibility of integrating the CBCT and DR systems into C‐ion RT workflows, fulfilling the requirement for precise target localization in carbon‐ion therapy. Our findings suggested that a slice thickness of 3 mm was clinically suitable for CT planning in C‐ion RT. Furthermore, a tube voltage of 120 kV was recommended as the default setting for routine CBCT and DR imaging to standardize imaging protocols.

## AUTHOR CONTRIBUTIONS

Yixiao Guo: Conceived and wrote original draft. Zhiqiang Liu, Qingzhen Zhu, and Ming Cai: Conceptualization, methodology, and data collection. Hongyi Cai and Ruifeng Liu: Discussed the results and commented the manuscript. Qiuning Zhang and Zhiguo Xu: Resources and review. All authors have read and approved the final manuscript.

## CONFLICT OF INTEREST STATEMENT

The authors declare no conflicts of interest.

## Supporting information



Supporting information
