# Supplementary material for: Evaluation and implementation of an independent Kilovoltage X‐ray‐based imaging platform for carbon ion radiotherapy
Source: J Appl Clin Med Phys. 2026 Feb 9;27(2):e70501. doi: 10.1002/acm2.70501 (PMC12885751; doi:10.1002/acm2.70501)
Supplement: Supplementary file 1 — Supporting information [file ACM2-27-e70501-s001.docx]

**Supplementary materials**

**Coincidence of imagi****ng and treatment isocenter**


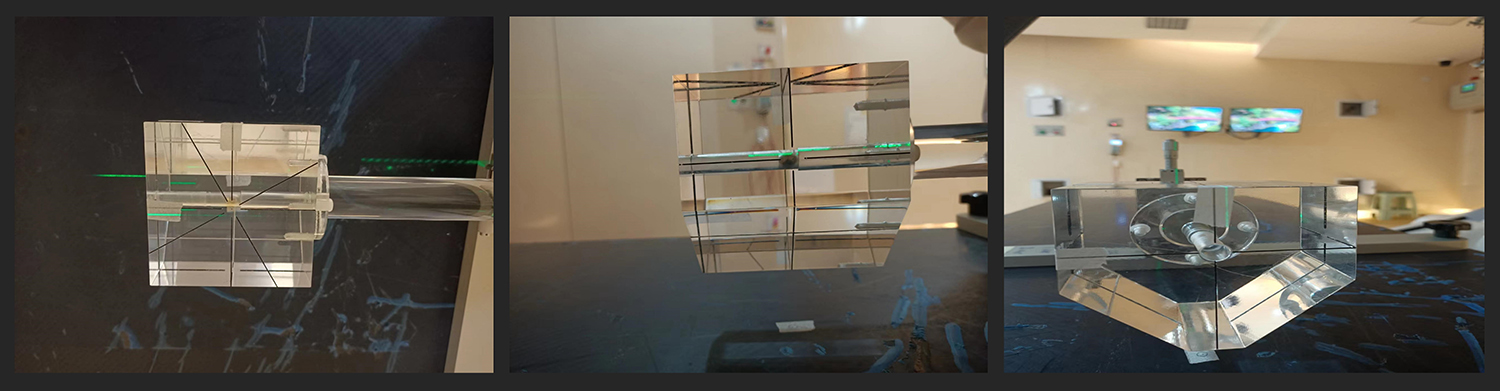


**FIGURE S-1.jpg** Congruence testing of imaging and treatment isocenters.

A specialized phantom, precisely aligned with room lasers, is employed to assess the positional relationship between the imaging isocenter and the treatment isocenter (Figure 3). The phantomʼs center is marked by crosshairs, representing the mechanical isocenter. The congruence of the imaging and carbon-ion beam isocenters is evaluated by comparing the radiopaque markers on the phantom with the center of the cone-beam computed tomography (CBCT) and/or orthogonal kilovoltage (kV) images acquired.

**TABLE** **S-1.docx** Technical specifications pertaining to the carbon ion facility in Heavy Ion Research Facility in Lanzhou

| Parameter | Indices |
| --- | --- |
| Particle species | ^12^C^6+^ |
| Treatment room | 4 |
| Accelerating method | High frequency electric field synchrotron |
| Port (gantry) type | Fixed port (gantry) |
| Fixed port (gantry) angle | Room 1：Fixed horizontal port (90°)  Room 2：Fixed horizontal and vertical ports (0° and 90°)  Room 3：Fixed vertical port (0°)  Room 4：Fixed 45° port |
| Beam delivery methods | Room 1 spot scanning  Room 2 uniform scanning  Room 3 spot scanning  Room 4 uniform scanning |
| Switching times for different treatment rooms | ＜60 s |
| Energy switching time | ＜60 s |
| Beam extraction time | 1s～10 s |
| Beam intensity range | 1×10^8^ ppp～4×10^8^ ppp |
| Energy range | 120～400 Mev/u |
| Maximum range | 280 mm |
| Energy regulation step | 1 MeV/u～5 MeV/u |
| Energy regulation methods | 1. Uniform scanning: discontinuous range conversion, the equipment is range shifter and ridge filter 2. Spot scanning: active energy change and micro-ridge filter |
| Field sizes | Room 1：≤200 mm×200 mm  Room 2：≤100 mm×100 mm  Room 3：≤200 mm×200 mm  Room 4：≤200 mm×200 mm |
| Dose rate | 0.001～1 Gy/s |
| Spot size (spot scanning) | ≤1.5 cm |
| Penumbra width (uniform scanning) | For nominal energy 260 MeV/u～400 MeV/u, the penumbra width ≤20 mm |
| Beam position accuracy | ±1 mm |
| The reference point position of the device | The most downstream scanning magnet mechanical center axis 6250 mm |
| Distance from the virtual source to the device reference point | Virtual source-axis distance ≥6.0 m |
| Single cycle minimum energy regulation step | 2 MeV/u |
| Maximum field size | 20 cm × 20 cm |

**TABLE** **S-2.docx** Technical specifications of six degrees of freedom couch

| Parameters |
| --- |
| Range of mechanical motion  X axis：±25 cm  Y axis：0 cm～88 cm  Z axis：-50 cm～20 cm  Z axis rotation：±180° |
| Weight  Couch load:135 kg (Room 1 and 2); 150 kg (Room 3 and 4). The weight is evenly distributed in the range of 2 m of the couch surface, and the gravity center acts on the isocenter point. The change of height near the isocenter of the couch under the above two loads ≤±1 mm  The maximum deviation between the rotation axis of the treatment couch and the treatment head: ≤ 0.2 cm (Room 1 and 2); ≤ 0.5 cm (Room 3 and 4)  The lateral inclination angle of the treatment couch surface relative to the horizontal plane: ≤ 0.5°  When the maximum transverse displacement of the treatment couch surface is made, the change of the height of the treatment couch surface near the isocenter ≤ 0.5 cm  Isocentric axis rotation error: ≤ ± 0.5° (Room 1 and 2); ≤ ± 0.2° (Room 3 and 4) |
| Motion control speed  The X /Y /Z axis moves in a straight line:1.0 cm/s, 4.0 cm/s  Isocentric rotation:1.0°/s, 4.0°/s |
| Stiffness  Lateral stiffness: ≤ 0.5 cm  Longitudinal stiffness: ≤ 0.5 cm |
| Motion range of the treatment couch  X axis：± 25 cm (Room 1 and 2)；-210 mm～+300 mm (Room 3 and 4)  Y axis：0 cm～88 cm (Room 1 and 2)；-450 mm～+350 mm (Room 3 and 4)  Z axis：-50 cm～20 cm (Room 1 and 2)；-300 mm～+200 mm (Room 3 and 4)  Z axis rotation：±180° (Room 1 and 2)；-5°～185° (Room 3 and 4)  Isocentric rotation range：200° (Room 1 and 2); -5°～185° (Room 3 and 4)  Casting angle: the left and right rotation angle of the couch plate is -5°～5° (Room 3 and 4)  Roll angle: the front and back tilt angle of the couch plate is -5°～5° (Room 3 and 4) |

**TABLE S-3.docx** Specification parameters of the CBCT and DR

| Image Guided Radiotherapy positioning system (Radsnipe XDR)  Model：Radsnipe con CT Production enterprises: Shinetone Medical Equipment Co., Ltd.  Software version number：V1.00.00.01  Registration method: bone, fiducial marker, grayscale  Fusion display modes: overlap, checkerboard, segmentation, lens | |
| --- | --- |
| DR image guidance system | CBCT image guidance system |
| Group A beams：  Distance from flat detector to isocenter: 730 mm±50 mm (Room3);  553.5 mm±50 mm (Room 4)  Distance from focus to detector：2680 mm±50 mm(Room3); 2103.5 mm±50 mm (Room 4)  Distance from focus to isocenter：1950 mm±50 mm (Room3); 1550 mm±50 mm (Room 4)  Group B beams:  Distance from flat detector to isocenter: 730 mm±50 mm (Room3) ;  553.5 mm±50 mm (Room 4)  Distance from focus to detector：2680 mm±50 mm (Room3);1953.5 mm±50 mm (Room 4)  Distance from focus to isocenter：1950 mm±50 mm (Room3);1400 mm±50 mm (Room 4)  Motion range from the center of the image detector to the X-ray beam axis：No movement (Room 3 and 4) | Distance from focus to detector：1053 mm (±50 mm)～580 mm(±50 mm)  Distance from focus to isocenter：580 mm(±50 mm)  Rotation range：-125° (±5°)～125° (±5°) |
| Angle between Group A and B beams: 90°(±5°) |  |
| Mechanical range of motion (up and down range):800 (±50 mm) |  |
| High voltage generator(Polydoros RF 80 OEM)  Tube voltage range：40 kV～150 kV  Tube current range：10 mA～1000 mA  Loading time range：20 ms～5000 ms  Nominal electrical power：80 kW (100 kV，800 mA，0.1 s)  Input supply voltage：AC380 V±10%  Power frequency：50 Hz±1 Hz  Current time product range:0.5～800 mAs  Maximum operating frequency:450 kHz  Power source internal resistance:≤0.11 Ω  Power capacity:≥90 kVA  High-frequency transformer structure：High frequency | High voltage generator (Polydoros RF 80 OEM)  Tube voltage range：40 kV～150 kV  Tube current range：10 mA～1000 mA  Tube current range：20 ms～5000 ms  Nominal electrical power：65 kW(100 kV，650 mA，0.1 s)  Input supply voltage：AC 380 V±10%  Power frequency：50 Hz±1 Hz  Current time product range: 0.5～800 mAs  Maximum operating frequency:450 kHz  Power source internal resistance: ≤0.11 Ω  Power capacity:≥90 kVA  High-frequency transformer structure：High frequency |
| X-ray tube assembly(SV_150_40_80 C_100_100L )  Nominal tube voltage：150 kV  Power: 40/80 kW(small focus/large focus)  Focus size：0.6 mm/1.0 mm(Small/large focus)  Target Angle：12°  Anode type:rotating anode  Maximum heat capacity of anode：450 kJ (600 kHU)  Maximum heat capacity of X-ray tube assembly：1800 kJ(2430 kHU) | X-ray tube assembly(SV_150_40_80 C_100_100L)  Nominal tube voltage：150 kV  Power: 40/80 kW(small focus/large focus)  Focus size：0.6 mm/1.0 mm(small focus/large focus)  Target Angle：16°  Anode type:rotating anode  Target Angle：450 kJ(600 kHU)  Maximum heat capacity of X-ray tube assembly：1800 kJ(2430 kHU) |
|  | Fixed beam limiter (M05)  Maximum X-ray area: 42.6 cm×42.6 cm (SID 1 cm )  Application scope: Target angle16°  Light projection region: No light |
| Bulb rated KV value  mA value range:10-800 mA  mAs value range:0.1-1000 mAs  Exposure time: 1-6000 ms | Bulb rated KV value  mA value range:10-800 mA  mAs value range:0.1-1000 mAs  Exposure time: 1-6000 ms |
| Flat panel detector  Maximum visual field：427.008 mm×427.008 mm  Material: amorphous silicon  Pixel size: 3072×3072 pixels (1×1); 1536×1536 pixels (2×2)  The maximum frame rate: 20 frames/s(2×2)  The minimum detector frame time: 50 ms | Flat panel detector  Maximum visual field：427.008 mm×427.008 mm；  Material: amorphous silicon  Pixel size: 3072×3072 pixels (1×1); 1536×1536 pixels (2×2) |
| Dynamic flat panel detector (Mercu1717 V)  Dynamic range: >77 dB @PGA6 1×1  Size: 470 mm×470 mm×35 mm  Detection area: 427.008 mm×427.008 mm  The visual field of the detectorʼs largest rectangular field (measured in the detector) : 43 cm×43 cm  Pixel matrix: 3072×3072 Pixel  Pixel:139 um  A/D conversion:16 bits  Maximum frame rate:10 fps (binning1×1, 3072×3072, 43 cm×43 cm FOV) | Dynamic flat panel detector (Mercu1717 V)  Dynamic range: >77 dB @PGA6 1×1  Size: 470 mm×470 mm×35 mm  Detection area:427.008 mm×427.008 mm  Pixel matrix:3072×3072 pixel  Pixel:139 um  A/D conversion:16bits  Maximum frame rate:10 fps (binning1×1, 3072×3072, 43 cm×43 cm FOV) |

**TABLE S-4.docx** Mechanical specifications of the support structure for KV planar X-ray image verification system

| Parameters |
| --- |
| Image detector for X-ray image positioning verification equipment  Room 1 ( 90°port)  The distance between the detector plane and the isocenter：260 mm-460 mm±5 mm (Image detector at 180°); 265 mm-465 mm±5 mm (Image detector at 270°)  The motion range from the center of the image detector to the X-ray beam axis：0-465 mm±5 mm  The displacement between the image detector center and the X-ray beam axis projection ≤ 0.5 cm (Image detector is at 180°and the distance from the source to the detector is 1055.0 mm)  The displacement between the image detector center and the X-ray beam axis projection: ≤0.5 cm (Image detector is at 270°and the distance from the source to the detector is 1060.0 mm)  Room 2 (0 and 90°ports)  The displacement between the image detector center and the X-ray beam axis projection: ≤0.5 cm (Image detector is at 180°and the distance from the source to the detector is 1055.0 mm)  The displacement between the image detector center and the X-ray beam axis projection: ≤0.5 cm (Image detector is at 270°and the distance from the source to the detector is 1100.0 mm) |
| Detector area  The detector effective area: 34.895×42.525 cm^2^  Effective imaging region  The nominal imaging area size: 427×427 mm^2^, the actual effective field of view should ≥95% of the nominal size |
| Detector Features  The distance between the X-ray image positioning verification device detector and the electronic reference point (ERP) is adjustable. The measured field of view ranges from a minimum of 34.8 cm×40.3 cm to a maximum of 34.8 cm×42.5 cm. |
| Location of the image detector  A consistent and specified distance between the detector plane and the isocenter is maintained for each measurement, ensuring uniformity across all assessments of the performance metrics of the electronic imaging equipment. |
| Visible pixel  The pixel matrix of the largest rectangle: 1,994×2,430 pixels |
| Detector frame time  Minimum image preview time: 2 s, A/D conversion: 16 bit |
| Signal-to-noise ratio and dynamic range of imaging system  Quantum detection efficiency (DQE) : ≥ 28 %(70 kV, 1.2 mAs, SID 100 cm, and the air specific kinetic energy at the image receiving surface ≤25 μGy) |
| Imager linearity: ≤ 78 dB |
| Incident exposure  Head: ≤ 5 mGy. 75 kV, 20 mAs, SID 110 cm (orthotopic);75 kV, 20 mAs, SID 110 cm (lateral)  Chest:≤ 10 mGy. 105-112 kV, 3-4 mAs, SID 110 cm (orthotopic);110-114 kV, 3-4 mAs, SID 110 cm (lateral )  Pelvic: ≤ 5 mGy. 84 kV, 32 mAs, SID 110 cm  Limbs:≤ 5 mGy. 72 kV, 6.3 mAs, SID 110 cm (knee); 68 kV, 5 mAs, SID 110 cm (joint); 65 kV, 5 mAs, SID 110 cm (upper limb) |
| Double X-ray imaging exposure interval  Imaging time: ≤ 8 seconds  Preview time:≤ 2 seconds |
| Image uniformity: the loading parameters are set at 50 kVp, 50 mA, and 2.5 mAs, and the ratio of the standard deviation (R) to the mean (Vm) of the gray values at the specified sampling point within the image should not exceed 2.2%. |

**Carbon-ion facilities in the Heavy Ion Research Facility in Lanzhou (HIRFL)**

HIRFL is equipped with an Electron Cyclotron Resonance (ECR) ion source, a 1.7-meter Sector Focused Cyclotron (SFC, K=69), a large Sector-Separated Cyclotron (SSC, K=450), and several designed therapy terminals. It has successfully accelerated carbon ions to energies between 120 and 400 MeV/u.


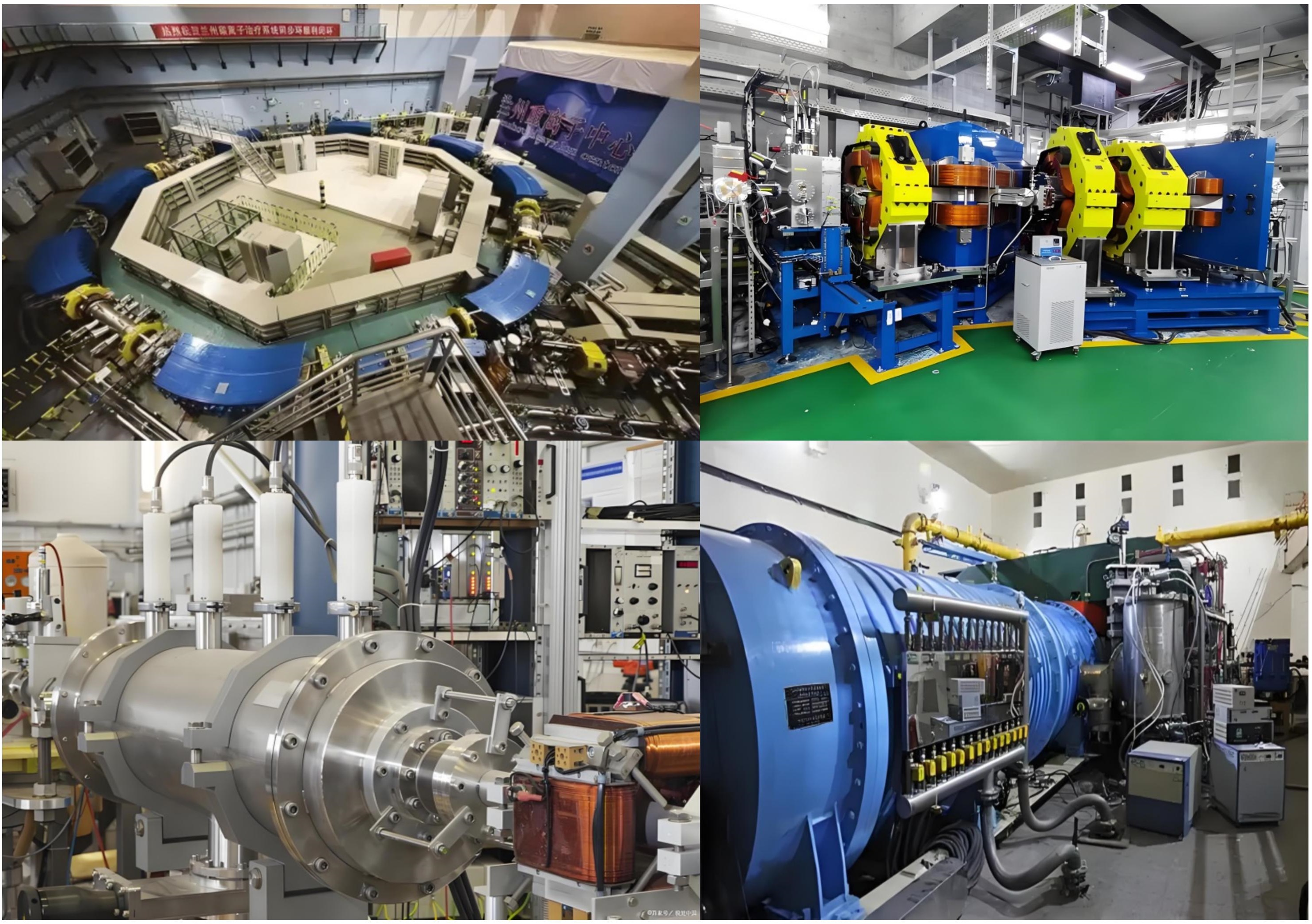


**FIGURE S-2.jpg** Pictures showing the carbon ion facility.

**CTP 714 high resolution module of the Catphan 700 phantom**

The 30 line pairs per centimeter gauge was employed for the visual assessment of the reconstructed spatial resolution. This gauge is fabricated from a 2 mm thick aluminum sheet and encapsulated in clear urethane. By using the rotation mount, the phantom can be oriented such that the gauges are either aligned with one of the scanner axes or positioned at 45-degree angles to the axis. The CTP 714 module comprises a series of cylindrical rods with varying diameters and three distinct contrast levels.


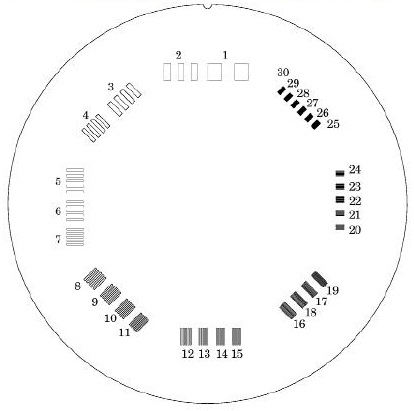


**FIGURE** **S-3.jpg** CTP 714 high resolution module with 1 to 30 line pairs per cm gauges.

**TABLE S-5.docx** Standards for reconstructing resolution.

| Line Pair/cm | Gap Size (cm) | Line Pair/cm | Gap Size (cm) | Line Pair/cm | Gap Size (cm) |
| --- | --- | --- | --- | --- | --- |
| 1 | 0.500 | 11 | 0.045 | 21 | 0.024 |
| 2 | 0.250 | 12 | 0.042 | 22 | 0.023 |
| 3 | 0.167 | 13 | 0.038 | 23 | 0.022 |
| 4 | 0.125 | 14 | 0.036 | 24 | 0.021 |
| 5 | 0.100 | 15 | 0.033 | 25 | 0.020 |
| 6 | 0.083 | 16 | 0.031 | 26 | 0.019 |
| 7 | 0.071 | 17 | 0.029 | 27 | 0.0185 |
| 8 | 0.063 | 18 | 0.028 | 28 | 0.0178 |
| 9 | 0.056 | 19 | 0.026 | 29 | 0.0172 |
| 10 | 0.050 | 20 | 0.025 | 30 | 0.0167 |

**Testing procedures for spatial resolution and low-contrast resolution**

A multi-purpose imaging phantom was employed to assess the in-plane spatial resolution of CBCT and DR images. The source-to-image distance (SID) was calibrated to align with the standard distance used in clinical practice. The image field of view was configured to the maximum effective imaging area of the detector plate. The manufacturerʼs recommended clinical protocol was employed, with the X-ray tube voltage manually set to 75 kV±7 kV, and exposure was conducted automatically. Following image acquisition, the original image was opened using the ImageJ software (with the raw file located in the original file directory) to analyze the maximum value of the three white lines within various line groups in the spatial resolution module. In the region containing 12 low-contrast holes, each with a diameter of 10 mm, the number of discernible circular holes was counted, facilitating the determination of low-contrast resolution.

**Positioning accuracy**

An anthropomorphic phantom (Chengdu Dosimetric Phantom, CPET Co. Ltd, China) was employed with five radiopaque fiducial markers to commission IGRT. A central reference marker P_0_ (x_0_, y_0_, z_0_) was positioned at the geometric isocenter of the phantom. In contrast, four non-coplanar peripheral markers (P_1_–P_4_: xᵢ, yᵢ, zᵢ, i = 1–4) were placed at predetermined offsets from P_0_ to delineate target volume boundaries. The head and neck and thoracoabdominal phantom sections were scanned sequentially in the supine position using a Somatom Sensation Open CT scanner (Siemens Medical Solutions, Erlangen, Germany). The planned CT images were transferred to the ciPlan treatment planning system (Institute of Modern Physics, Chinese Academy of Sciences, Lanzhou, China), with P_0_ (x_0_, y_0_, z_0_) designated as the tumor target. Skin marks were aligned with the internal planning isocenter, which served as the reference point for subsequent image registration. These CT datasets were exported to the IGRT workstation as reference images for CBCT- and DR-based registration. Then, the phantom was positioned on the carbon-ion treatment couch with P_0_ aligned to the treatment isocenter F_0, 0_ (0, 0, 0). The spatial congruence between the phantomʼs geometric center and the planned isocenter was verified using volumetric scanning, which was defined as the measurement origin. Following the initial alignment, the actual target coordinates, denoted by R_0, 0_ (x_0, 0_, y_0, 0_, z_0, 0_), were recorded. To simulate clinical displacements, the phantom was translated along three anatomical axes (right, caudal, and posterior) to a predefined offset position F_0, 1_ (d, d, d), where d = 10 mm for the head-neck region, and 15 mm for the thoracoabdominal region. Post-translation, the actual target coordinates R_0, 1_ (x_0, 1_, y_0, 1_, z_0, 1_) were documented. Then, registration software was used to calculate the displaced target coordinates R'_0, 1_ (x'_0, 1_, y'_0, 1_, z'_0, 1_) relative to the isocenter. The spatial displacement between the calculated target coordinates R'_0, 1_ (x'_0, 1_, y'_0, 1_, z'_0, 1_) and the actual displaced position R_0, 1_ was quantified using Equation (1), thereby evaluating the ability of the IGRT system to compensate for translational shifts.

${\Delta r}_{0,1}=\sqrt{\left( x_{0, 1}^{'}-x_{0, 1} \right)^{2}+\left( y_{0, 1}^{'}-y_{0, 1} \right)^{2}+\left( z_{0, 1}^{'}-z_{0, 1} \right)^{2}}$ (1)


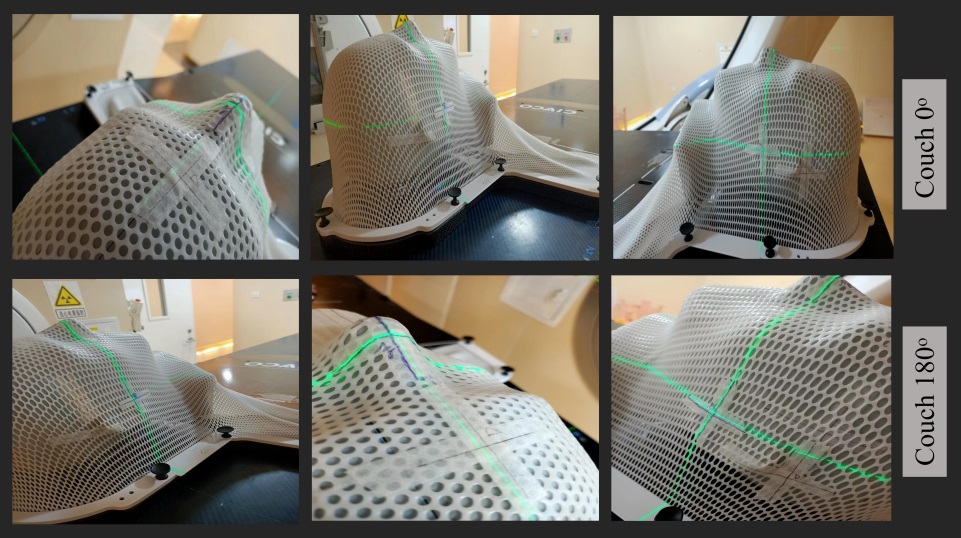


**FIGURE S-4.jpg** A male anthropomorphic head-neck phantom was placed on a six-degree-of-freedom positioning system (couch 0^o^ and 180^o^) and imaged to evaluate the image guidance accuracy.


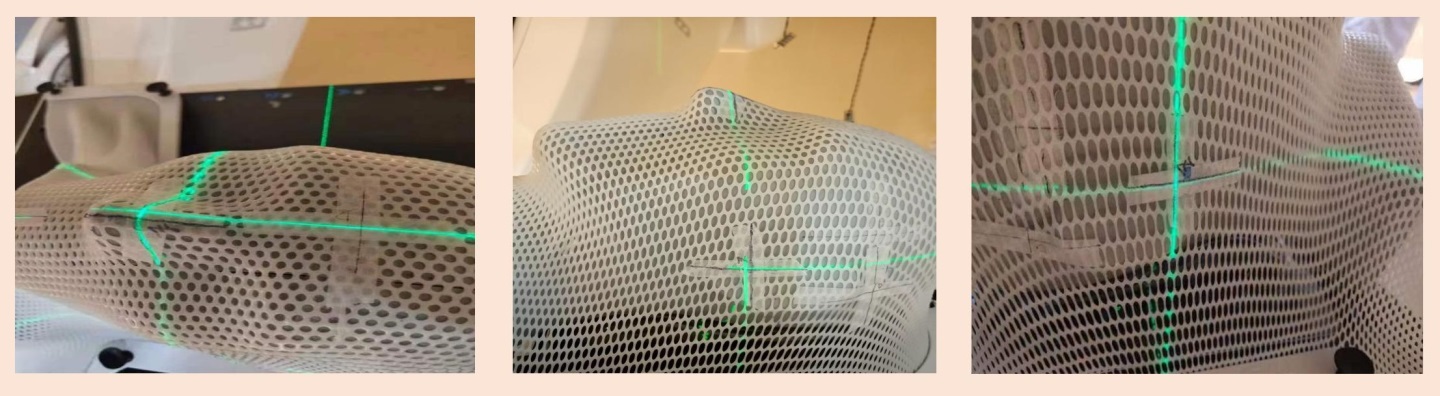


**FIGURE** **S-5.jpg** A 5 mm deviation is artificially introduced in the three lateral directions, and the images are acquired for position correction (couch 0^o^).


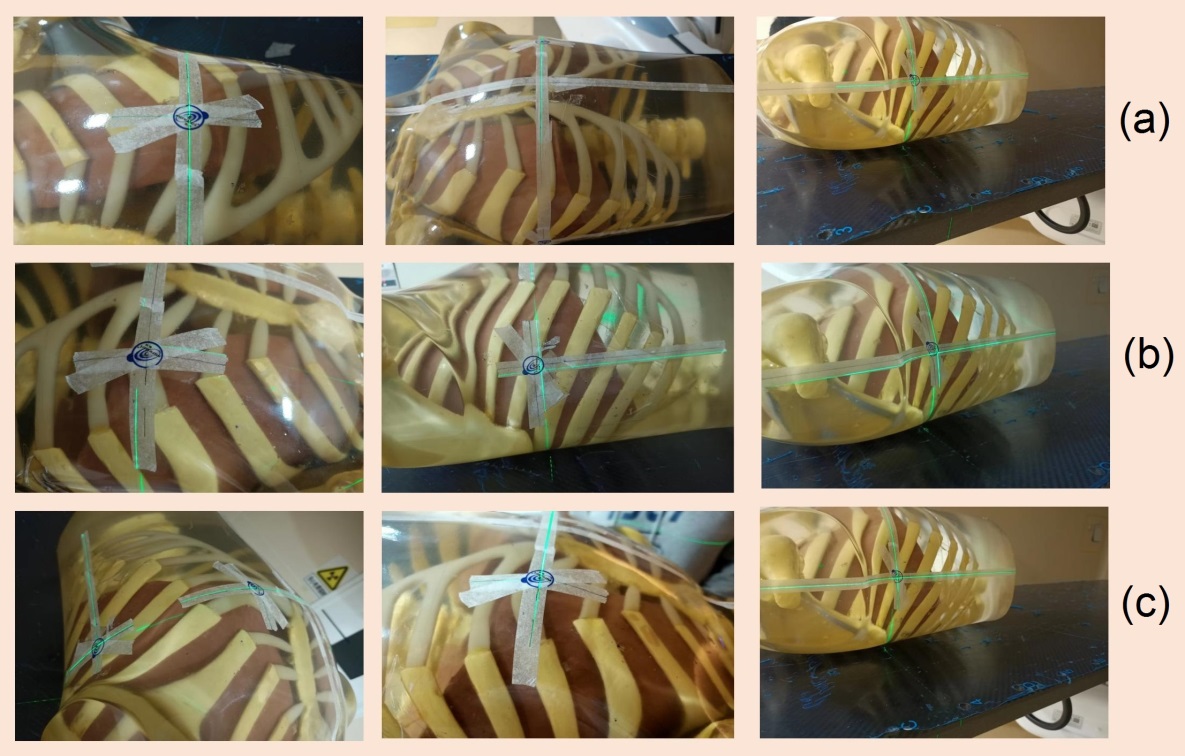


**FIGURE** **S-6.jpg** (a) An anthropomorphic thoracoabdominal phantom was imaged to assess the image guidance accuracy. (b) A 5 mm deviation was deliberately introduced in the three lateral directions. (c) Following error correction, the residual errors were evaluated.

**Protocol workflow for phantom testing**

1) Effect of reference image layer thickness

Head-neck phantom

a) The phantom was positioned on the couch and scanned using minimum, maximum, and reference layer thicknesses to acquire CT images.

b) Choose the CT image set with the smallest thickness.

c) Select a typical reference image reconstruction algorithm.

d) The acquired CT images were reconstructed, and the associated treatment plan was subsequently developed. Point P_0_ (x_0_, y_0_, z_0_) in the phantom is selected as the target.

e) By adjusting the phantom positioned on the couch, the target was aligned to the coordinates F_0_ (d, d, d). For the head-neck phantom, the value of d is specified as 10 mm.

Note: The coordinate system used here is consistent with the patient coordinate system in GB/T 18987.

f) Set the resolution of the IGRT imaging display device to a typical value.

g) Select the typical radiation quality of IGRT.

h) Initiate IGRT imaging.

i) For stereo-plane imaging IGRT, a typical reference image DRR algorithm is selected.

j) For CBCT device, the typical IGRT image reconstruction algorithm is selected for registration.

k) Calculate the coordinates F_0, min_ (x, y, z) of target relative to the isocenter (or radiotherapy reference point).

l) The CT image sets with both maximum and typical layer thicknesses were selected, and steps c), d), and f) ~ j) were repeated.

m) The coordinates F_0, max_ (x, y, z) and F_0, t_ (x, y, z) of the target relative to the isocenter (or radiotherapy reference point) are calculated, respectively.

n) Calculate the deviations between F_0, min_ (x, y, z) and F_0, t_ (x, y, z) and between F_0, max_ (x, y, z) and F_0, t_ (x, y, z), respectively.

Thoracoabdominal phantom

The method employed is consistent with those described in sections a) ~ n). It is important to note that, for the thoracoabdominal phantom, the parameter d is 15mm, Φ=100mm.

2) Influence of radiation quality on IGRT setup correction calculation

Head-neck phantom

a) The phantom was positioned on the couch and scanned in accordance with the standard scanning protocols outlined by the IGRT manufacturer to acquire CT images.

b) A typical reference image reconstruction algorithm was employed to reconstruct the acquired CT images, develop an associated treatment plan, and designate the point P_0_ (x_0_, y_0_, z_0_) within the phantom as the target.

c) The phantom was moved to the carbon-ion treatment couch for setup. By adjusting the test phantom, the target point was aligned to the coordinates F_0,0_ (d, d, d), where d = 10 mm.

d) Set the resolution of the CBCT or DR imaging display device to a typical value.

e) Choose the typical tube voltage.

f) Initiate IGRT imaging.

g) For stereo-plane imaging IGRT, a typical reference image DRR algorithm is selected, while the typical image reconstruction algorithm is utilized in CBCT system.

h) A typical image registration algorithm is selected for registration.

i) Calculate the coordinates F_0, t_ (x, y, z) of target relative to the isocenter.

j) For kV imaging, choose the applicable minimum and maximum tube voltages respectively, start IGRT imaging, repeat steps f) ~ i).

k) Calculate the coordinates F_0, min_ (x, y, z) and F_0, max_ (x, y, z) of target relative to the isocenter.

l) Calculate the deviations between F_0, min_ (x, y, z) and F_0, t_ (x, y, z) and between F_0, max_ (x, y, z) and F_0,t_ (x,y,z), respectively.

Thoracoabdominal phantom

The method employed is consistent with that described in sections a) ~ l).

3) Influence of image registration algorithm on setup errors

Head and thoracoabdominal phantoms

a) The phantom was positioned on the couch and scanned to acquire CT images.

b) A typical reference image reconstruction algorithm was employed to reconstruct the acquired CT images.

c) The test phantom was moved to the carbon-ion treatment couch for setup, and the target point was aligned to the coordinates F_0, 0_ (d, d, d).

d) Set the resolution of the X-IGRT imaging display device to a typical value.

e) IGRT imaging was initiated by selecting the typical radiation of IGRT.

f) For stereo-plane imaging IGRT, a typical reference image DRR algorithm is selected. For CBCT device, the typical reconstruction algorithm is utilized.

g) A typical image registration algorithm is selected for registration.

h) Calculate the coordinates F_0, t_ (x, y, z) of the target relative to the isocenter.

i) Select other registration algorithms.

j) The coordinates F_0, i_ (x, y, z) of the target relative to the isocenter are calculated, respectively, where i is the number of other image registration algorithms.

k) Calculate the distance between F_0, i_ (x, y, z) and F_0, t_ (x, y, z) respectively.


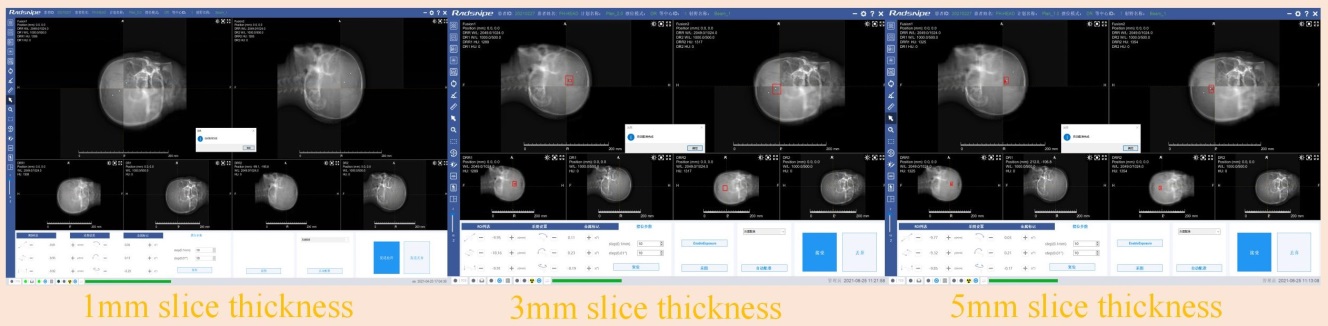


**FIGURE** **S-7.jpg** Registration interface diagram of different slice thickness for head phantom DR Imaging.


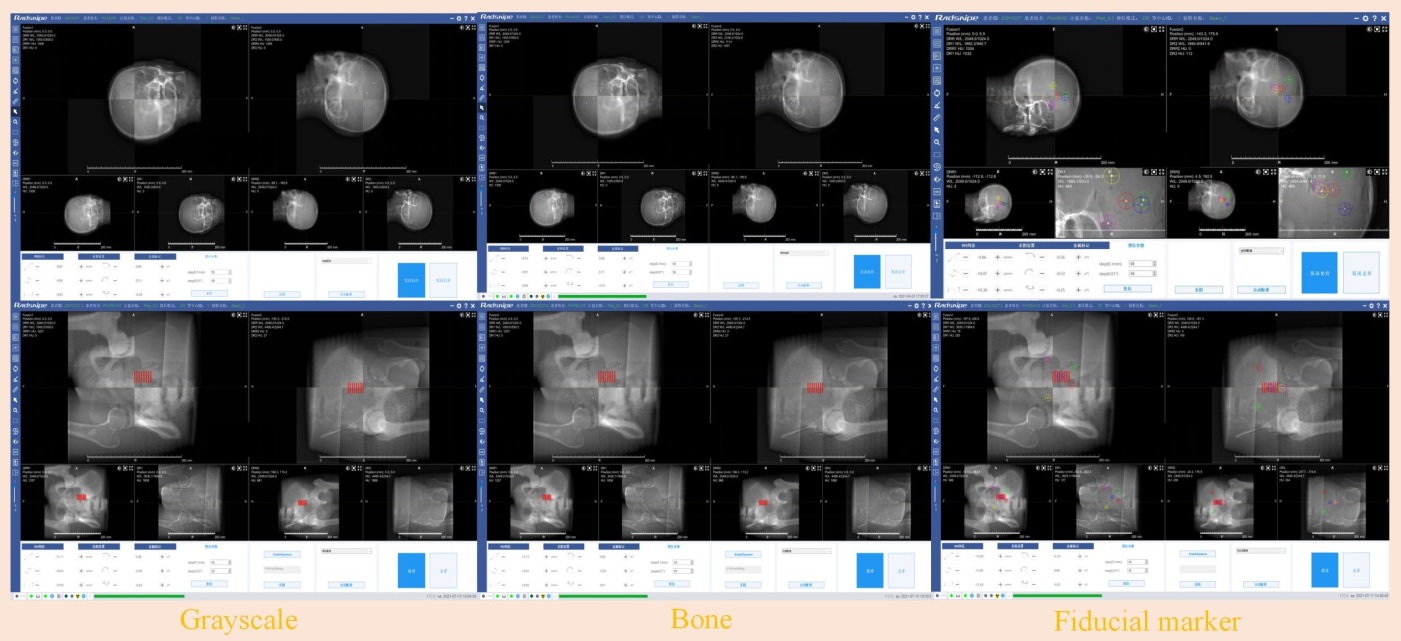


**FIGURE S-8.jpg** Interface diagrams for different registration algorithms in DR Imaging using anthropomorphic head-neck and thoracoabdominal phantoms.

Fusion visualizations are presented in Figures 10 and 11. This image-to-image fusion technique employs split-screen displays with native CBCT and reformatted CT images. The DR registration interface shows six windows, two of which display the sagittal and coronal views of the planning CT. Translational and rotational offsets were graphically overlaid onto CT images by applying geometric transformation matrices.


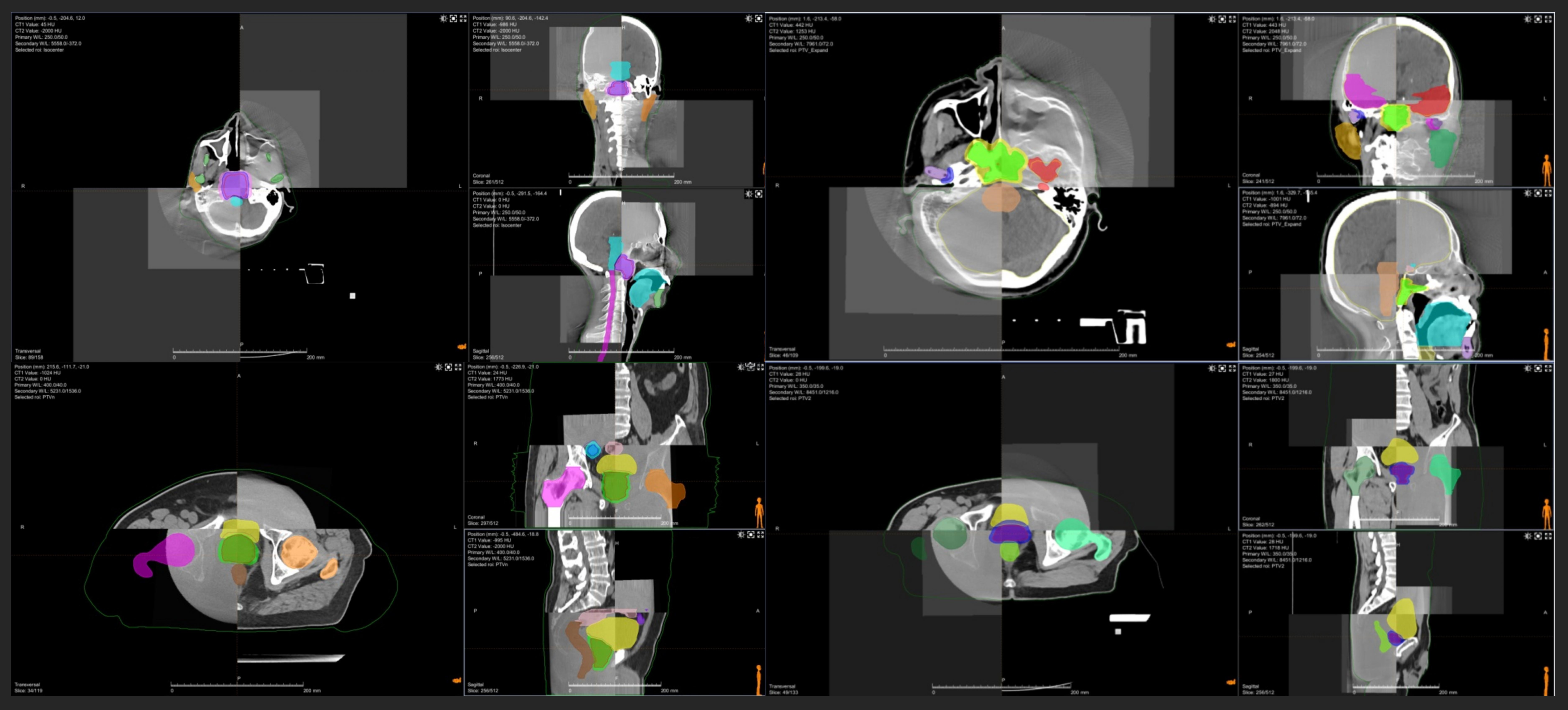


**FIGURE** **S-9.jpg** Illustration of axial, coronal, and sagittal CBCT-CT fusion images for representative head-neck and pelvic patients. The checkerboard patterns for the synthetic and CBCT images are presented.


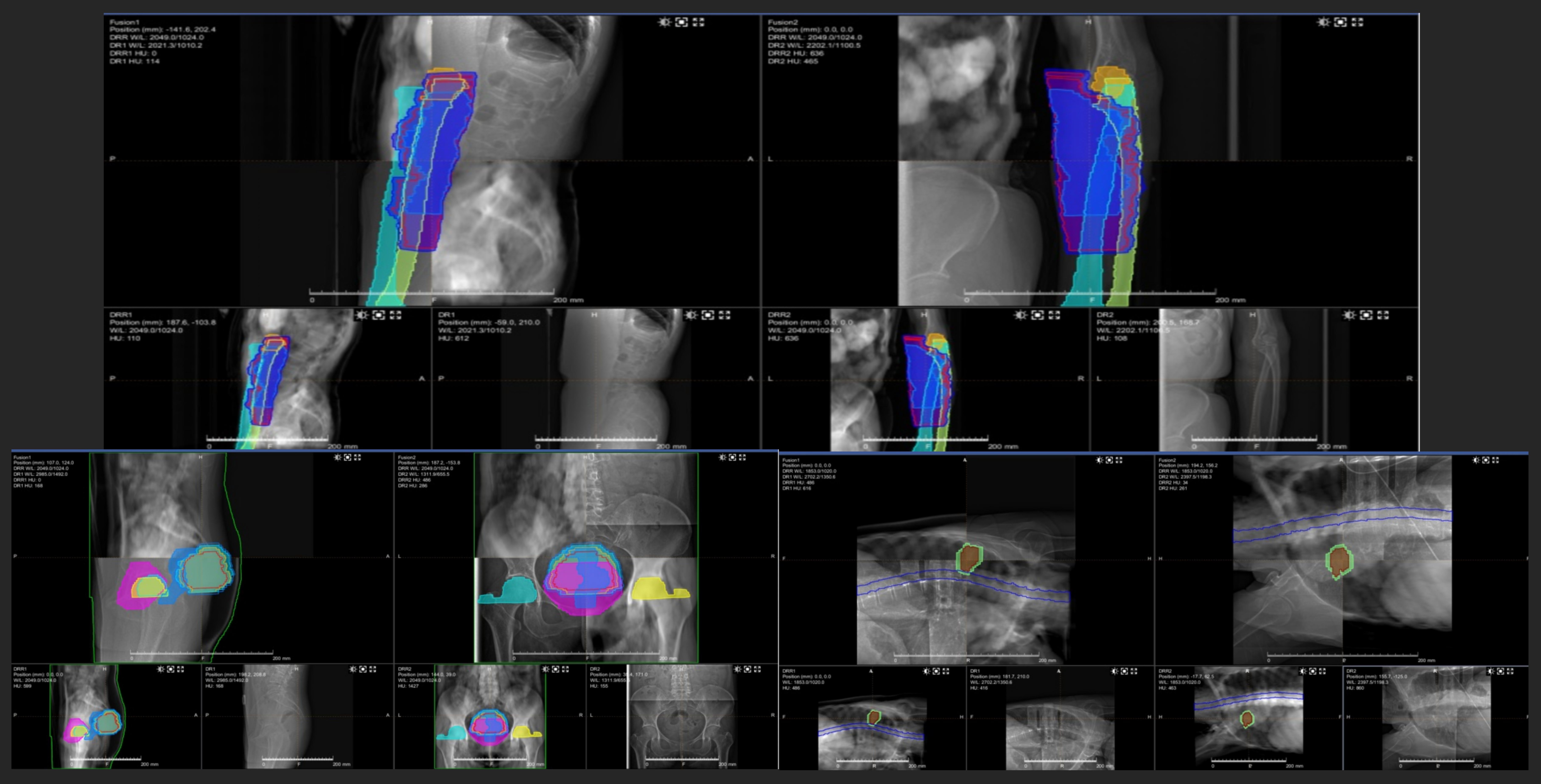


**FIGURE** **S-10.jpg** Visual comparison of orthogonal kV projections with DRR images of limb and abdominal patients.
